# The Lichtenberg Keilmesser - it’s all about the angle

**DOI:** 10.1371/journal.pone.0239718

**Published:** 2020-10-06

**Authors:** Marcel Weiss

**Affiliations:** Max Planck Institute for Evolutionary Anthropology, Leipzig, Germany; Universita degli Studi di Ferrara, ITALY

## Abstract

The presence of the ‘Keilmesser-concept’ in late Middle Paleolithic assemblages of Central and Eastern Europe defines the eponymous ‘Keilmessergruppen’. The site of Lichtenberg (Lower Saxony, Germany) was discovered in 1987 and yielded one of the most important Keilmessergruppen assemblages of the northwestern European Plain. At that time, researchers used the bifacial backed knives to define a new type, the ‘Lichtenberger Keilmesser’, which they characterized by an aesthetic form-function concept with a specific range of morphological variability on the one hand, and a standardized convex cutting edge one the other hand. Thereby, a shape continuum was observed between different form-function concepts in the Lichtenberg assemblage, from Keilmesser through to Faustkeilblätter and handaxes. In a contrasting view, it was recently suggested that the morphology of Keilmesser, including what is defined here as type Lichtenberg, is the result of solutions to establish and maintain edge angles during resharpening. With the intention to evaluate these contrasting hypotheses, I conducted a re-analysis of the Keilmesser from Lichtenberg and their relationship to central German late Middle Paleolithic knives, using 3D geometric morphometric analyses and an automatized approach to measure edge angles on 3D models. Despite a morphological overlap of the tools from both regions, I could show that the Lichtenberg Keilmesser concept refers to one solution to create a tool with specific functionalities, like potentially cutting, prehension, and reusability. To establish and maintain its functionality, certain angles where created by the knappers along the active edges. This behavior resulted in specific shapes and positions of the active parts and created what looks like a standardized or template morphology of this Keilmesser type.

## Introduction

The bifacial backed knife, and more specifically the Keilmesser-concept, observed on bifacial and unifacially shaped tools [1], is the most prominent tool type of the central European Micoquian [2–6]. Furthermore, its presence in late Middle Paleolithic (LMP) assemblages defines the eponymous Keilmessergruppen [7, 8]. Based on earlier definitions [3, 9–11], Jöris [12, 13] defines the tool as bifacial cutting tool with a working edge opposite an unworked or roughly worked back, a base in the proximal part adjacent to the back, as well as a second, sometimes also sharp edge in the distal part (distal posterior part) that converges with the cutting edge and forms an often pointed distal tip.

**Fig 1:**
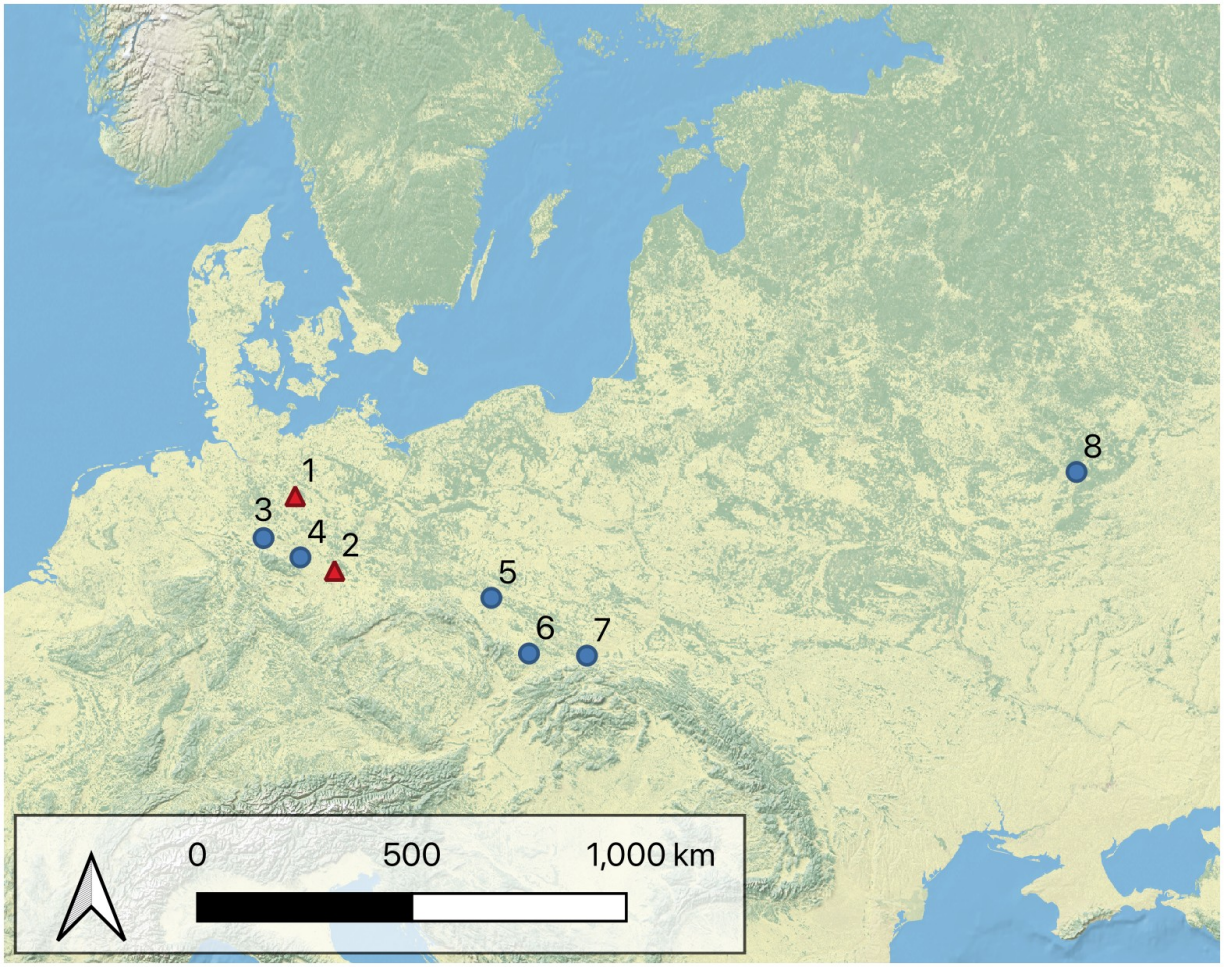
Map showing the sites mentioned in the text. Red triangles: assemblages included in the dataset. Blue dots: central to eastern European sites with comparable bifacial backed knives. 1: Lichtenberg; 2: Pouch, Löbnitz and Goitzsche; 3: Salzgitter-Lebenstedt; 4: Königsaue; 5: Wrocław-Hallera Av.; 6: Pietraszyn 49a; 7: Piekary IIa and Piekary III; 8: Khotylevo. Map created with QGiS 3.12, Basemap: Natural Earth (http://www.naturalearthdata.com/).

**Fig 2:**
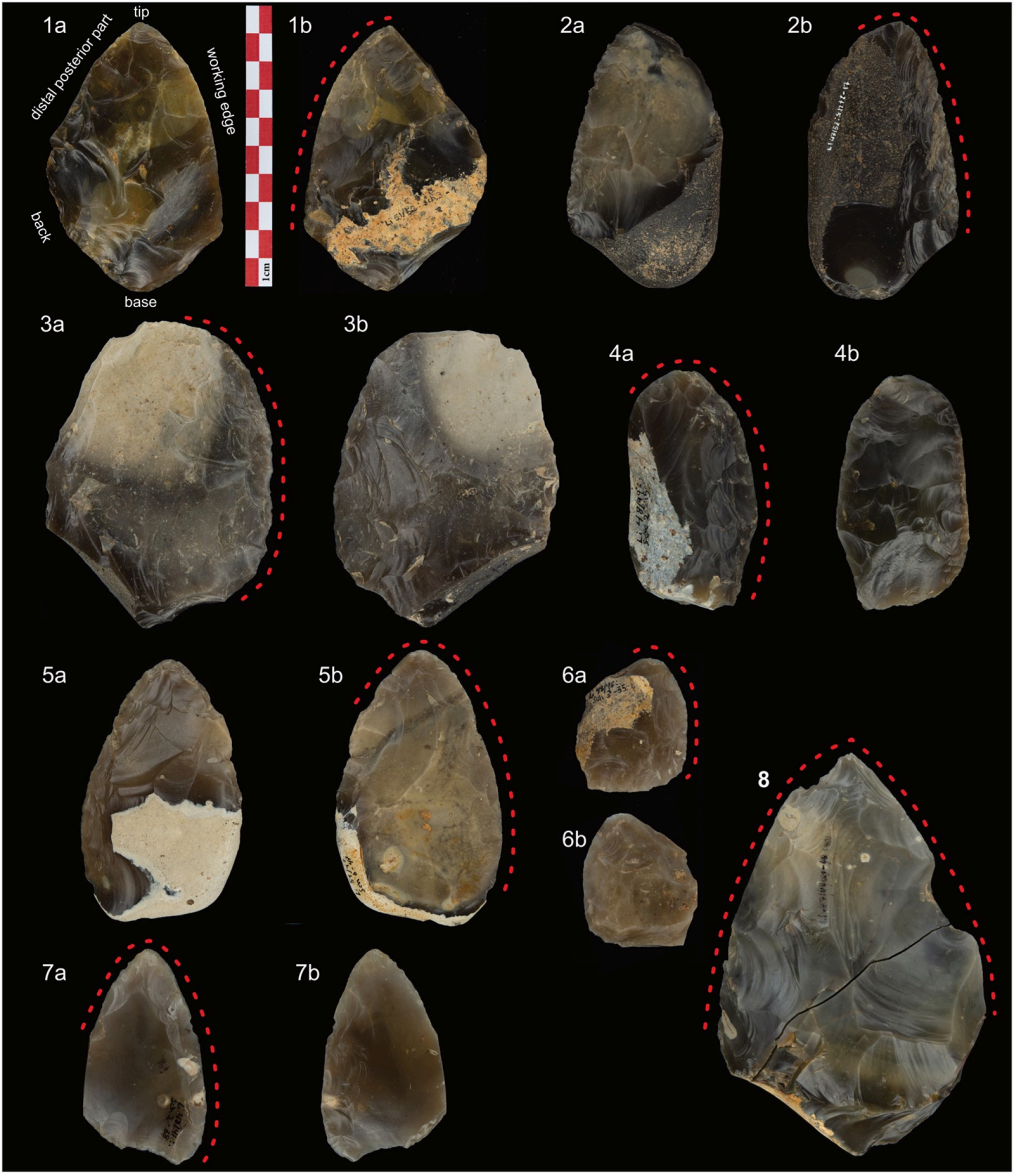
Examples of Keilmesser from Lichtenberg. The main techno-morphological parts are displayed in subfigure 1. Red dotted lines mark the extension of sharp edges. Note the difference of the extension of the second lateral working edge on Keilmesser (1-4 and 6), Faustkeilblätter (5 and 7) and handaxe (8). 1: Keilmesser with intentional break at the distal posterior part (51/52-3-1); 2: Keilmesser with cortical back and rounded tip (47/52-12-17); 3: Keilmesser with steep and rounded distal posterior part (51/45); 4: Keilmesser with rounded tip (48/49-2-15); 5: Faustkeilblatt or Keilmesser with long distal posterior part (54/48-6-47); 6: small Keilmesser with rounded tip (48/46-3-35); 7: Faustkeilblatt or Keilmesser with long distal posterior part and pointed tip (48/46-2-38); 8: Handaxe (47/49-40). Photos: MPI EVA.

When Veil et al. [8] discovered the site of Lichtenberg (Lower Saxony, Germany) in 1987 (Fig 1), they found one of the most important Keilmessergruppen assemblages of the northwestern European Plain. Veil [8] and Jöris [12, 13] used the bifacial backed knives to define a new type, the Lichtenberg Keilmesser (Fig 2). Veil [8, 14] describes the ideal tool as follows: an oval shaped outline with a longitudinal symmetry, especially in the tip region, a convex lateral working edge that extends to a retouched and often rounded sharp tip at the distal part, and a natural or partly retouched back. The back opposite a sharp working edge results in a wedge shaped cross section of the tool. Bifacial backed knives resembling Lichtenberg Keilmesser occur in several sites across the central and eastern European Plain between Marine Isotope Stage (MIS) 5a and MIS 3 (Fig 1). Examples are Salzgitter-Lebenstedt (Lower Saxony, Germany) [15, 16], Königsaue layer A and C (Saxony-Anhalt, Germany) [17, 18], Pouch (Saxony-Anhalt, Germany) [1, 19], Piekary IIa layer 7c [20] and Piekary III [21] (Poland), Wroław-Hallera Av. (Poland) [22], Pietraszyn 49a (Poland) [23], and Khotylevo (Russia) [24–28]. Potentially, comparable tools to the Lichtenberg Keilmesser occur as far east as southern Siberia [29].

From a morphological point of view, especially the handaxe-like oval outline shape, as well as the mostly rounded tip with a circumferential working edge extending to the distal posterior part make it different from other Keilmesser types (see e.g., figures in [12, 13]). However, within the range of variability are also pointed tips, differing form the ideal case [8] (Fig 2:1). Alongside the Lichtenberg Keilmesser exist other types of bifacial backed knives within the Keilmessergruppen [3, 12, 13]. But Jöris [12, 13] demonstrated the existence of a resharpening trajectory between them, and most of the types morphologically merge into one another. More importantly, there is a technological difference between Lichtenberg Keilmesser and Keilmesser resharpened with the tranchet blow (Keilmesser with tranchet blow, KMTB [30]) parallel along the working edge (see also [9, 12, 13, 31, 32] for further definition and explanation of the underlying concepts). Here, the distal end of the tool is prepared as striking platform for longitudinal resharpening removals directly along the working edge. This creates a sharp lateral working edge with practically one strike. Due to the performance of resharpening blows from the distal end [32], the KMTBs are also morphologically different from the Lichtenberg Keilmesser: The distal posterior part resembles here a highly convex bow without a circumferential working edge at the tip (that we see in the ‘ideal’ Lichtenberg Keilmesser) to create a striking platform perpendicular to the working edge for the longitudinal resharpening removals.

Within his type definition, Veil [8, 14] interprets the morphology of the Lichtenberg Keilmesser as a result of a special aesthetical form-funtion concept that Neanderthals had in mind during manufacture. For him, the concept of a relatively long back, a sharp tip, together with a longitudinal symmetry and a convex cutting edge of Keilmesser is conceptionally different to other tool categories within the Lichtenberg assemblage. However, according to Veil the most *standardized* element of Keilmesser is the convex cutting edge, which he also recognized on other (static) tool types, like “Faustkeilblätter”, handaxes, and leaf-shaped scrapers. Faustkeilblätter (Fig 2:5 and 2:7) are related to Keilmesser, as they have also a working edge opposite a back, a base, and a tip with a circumferential working edge. In contrast to the latter, their distal posterior part is relatively long (i.e., longer than the back) and resembles a rather thin edge. Handaxes (Fig 2:8) are characterized by a thin symmetric tip, formed by two lateral working edges, and an unworked base. One of the lateral edges can be slightly shorter than the other and is connected to a back-like extension of the base. Leaf-shaped scrapers are bifacial tools with an oval outline shape and a transversal symmetry. Based on their morphological difference to the tools mentioned before as well as their opposite symmetry concept, they are not included into the further analyses. Despite aesthetics, Veil interprets the overall shapes of the specific tool types as means to fulfill specific functional tasks. However, he admitted that morphological variability within and divergence from the ideal form-concepts exist in the assemblage. Veil [8] argues that this may be caused by resharpening or the pragmatic use of raw material features, like e.g., a Keilmesser-like shaped natural piece which was transformed through marginal retouch into a bifacial backed knife (Veil et al. 1994: 34 [8]).

Jöris [13] also characterized the Lichtenberg Keilmesser by a high overall morphological variability. He highlights as well the standardized convex cutting edge, which seems to be contrary to a stated high morphological variability of the tool. In contrast to Veil, Jöris [13] observed a shape continuum from Keilmesser through to Faustkeilblätter and handaxes.

But what does the “*high morphological variability*” mean and how is variability structured within this type? If the morphology is highly variable, what, in the end, constitutes the type or the form-function concept besides the retouched tip, the convex cutting edges and the lack of tranchet blows?

Contrasting Veil’s ideas of fixed form-function concepts, Iovita [33] hypothesized that angle reduction of the active edges is one of the main factors that drive LMP tool morphology and technological features. He states that the overall morphology of LMP tools is designed as a technical solution to handle the problem of increasing edge angles during use and subsequent resharpening. Further, it was suggested [19, 34, 35] and has been shown [1, 33, 36–38] that these concepts and related life histories apply likewise to unifacial and bifacial LMP tools. Following Iovita, there are three different solutions in the LMP to solve the problem of increasing edge angles: (1) thinning the tool volume using the back as striking platform (see also [11]), (2) the reduction of the edge angle through blows directly from the edge, and (3) the tranchet blow struck from the distal edge to thin the tool volume directly along the working edge [30, 32]. The first concept includes the distal posterior part, as Iovita did not separate this edge from the back. According to him, non-KMTB Micoquian bifacial backed knives, including the Lichtenberg Keilmesser, where manufactured and maintained using the first and second solution. If we follow the arguments made by Iovita [33], this would imply that the tool morphology is dictated by technological solution(s) to maintain an acute angle of the working edge during subsequent use of a long-living tool. On the other hand we have to be cautious, as subsequent resharpening can also alter the shape of tools [31, 33, 36, 37, 39–47]. Especially Keilmesser change overall shape and size during subsequent reduction [12, 13, 31, 37, 47–50]. But Iovita [37] could show that despite an allometric shape change during resharpening, the individual parts of Keilmesser from Buhlen (Hesse, Germany) change isometrically in relation to each other and the techno-functional and prehensile units stay constant on the tools (for a contrasting view on Keilmesser resharpening see e.g., Richter [49] and Uthmeier [50]). Summarizing Iovita’s ideas, he created the idea that the Keilmesser is designed for edge angle maintenance during subsequent resharpening, while the functional units stay constant.

Following from what I said above, there are two main hypotheses to explain the variability and morphology of the Lichtenberg Keilmesser: 1) it was a solution for maintaining acute edge angles on the active parts of a tool, or 2) it was a form-function template and an aesthetic design type.

The present study is an attempt to get new insights about the mechanisms and the structure of variability within this tool type. I am going to focus here on the analysis of Lichtenberg Keilmesser from the eponymous site (Fig 1). To increase sample size and to analyze the tools within a broader context, I incorporated my recently published data set [51] of late Middle Paleolithic Keilmesser from central Germany. The tools are also characterized by a convex cutting edge opposite a back, an often sharp distal posterior part and a retouched and mostly rounded tip, and match therefore the definition of the Lichtenberg Keilmesser.

Following a brief technological description, I performed a 3D geometric morphometric (3DGM) analysis in R [52–55], to draw inferences about Keilmesser shape variability.

To evaluate the assumed standardization of the working edge, I used the following approach: If we split the tool concept into morpho-functional units [16, 48, 56–60], it consists of a prehensile part, the base and the back, and two active edges, the working edge and the distal posterior part, including the distal tip formed by both edges. As the back and the base consist mostly of natural and/or roughly worked surfaces with an inherent natural variability, I assumed that the retouched active edges of the tool concept are the most important parts to trace mechanisms that structure tool variability. I therefore anaylzed separately the 3D geometry of the distal posterior part and the back on the one hand, and the working edge on the other hand to see which parts are the most variable.

In the next step, I applied an automated approach to measure edge angles on 3D models [53]. With this method I was able to conduct a detailed edge angle analysis of the active edges to evaluate the ideas brought forward by Iovita [33], and to get insights about edge function. Finally, I used the edge angle and 3DGM data to analyze the reduction and resharpening of the Keilmesser within my dataset.

The combined approach of technological observations, 3DGM and automated edge angle analysis obtained from 3D models aims to provide new insights into the structure of variability underlying a dataset of central European LMP knives and the meaningfulness of their classification as a special type.

## Materials

### Artifacts

The sample of tools from Lichtenberg analyzed in the present study are stored in the *Landesmuseum Hannover, Das Weltenmuseum, Willy-Brandt-Allee 5, 30169 Hannover, Germany*. The permission to study the material was granted with a cooperation contract between the *Max Planck Institute for Evolutionary Anthropology, Deutscher Platz 6, 04103 Leipzig, Germany, Dept. of Human Evolution* and the *Landesmuseum Hannover*. All necessary permits were obtained for the described study, which complied with all relevant regulations. The assemblage of Pouch and parts of the collection from Goitzsche are stored in the *Landesamt für Denkmalpflege und Archäologie Sachsen-Anhalt—Landesmuseum für Vorgeschichte Richard-Wagner-Straße 9, 06114 Halle (Saale), Germany*. The second part of the assemblage Goitzsche, as well as the collection from Löbnitz are stored in the *Landesamt für Archäologie Sachsen, Zur Wetterwarte 7, 01109 Dresden, Germany*. The datasets from Pouch, Löbnitz and Goitzsche have been already published by the author within four articles and his dissertation [1, 19, 51, 61, 62] and required no additional permits, which complied with all relevant regulations. The numbers of the individual specimens are provided within the text, the .RData file within the Supplementary Information to recreate the article with rMarkdown, as well as in the S1 Table.

I incorporated 35 bifacial Keilmesser and 7 unifacially shaped Keilmesser (Table 1) from the sites Lichtenberg, Pouch, Löbnitz and Goitzsche (Fig 1) in my dataset. The sample size for Pouch (6) and Goitzsche (3) is low, but the focus of the present study lies mainly on the Lichtenberg Keilmesser type and not on a comparison of tools from different sites. In the few cases that I do that the results have to be regarded as tentative. I included also 4 handaxes (Fig 2:8) as a morphological out group to test the reliability of the 3D geometric morphometric analysis. Although the late Middle Paleolithic handaxes are interpreted as related to Keilmesser [63, 64], their two working edges and their overall symmetrical shape result in a morphology distinct from the asymmetric Keilmesser [1, 8, 13]. Due to their symmetric shape and narrow range of morphological variability [1] they should form their own group within the multivariate analyses. Furthermore, at least two of the Keilmesser from Lichtenberg could by typed as Faustkeilblätter (Fig 2:5 and 2:7) according to Veil [8, 14]. I included them because regardless of their longer distal posterior part, they share the main techno-morphological elements with Keilmesser. Further, their inclusion may help to evaluate Jöris’ hypothesis of a continuum between Keilmesser, Faustkeilblätter and handaxes.

**Table 1:** Sites and artifacts included in the dataset.

Site	bifacial Keilmesser	unif. shaped Keilmesser	Handaxes	Date
Lichtenberg	19	3	1	66±14.6 ka—52±6.8 ka
Pouch	3	3	0	47.1±2.7 ka, 46.2±2.5 ka
Löbnitz	11	0	3	early MIS 3
Goitzsche	2	1	0	early MIS 3

References for the dates are given in the text.

### Assemblages

The site of Lichtenberg was discovered in 1987 and subsequently excavated by the *Landesmusem Hannover* until 1993 [8, 14]. The assemblage contained 405 artifacts with recorded provenience, among them 76 retouched tools. The numerical age was measured using thermoluminescense. Dating uncertainties place the assemblage between MIS 5a and early MIS 3. The thermoluminescence age range from 66±14.6 ka to 52±6.8ka [8]. I incorporated a sample of 19 bifacial Keilmesser, 3 unifacial Keilmesser and one handaxe (Table 1) into the present study. All artifacts are preserved in fresh condition.

The site of Pouch [1, 19, 51, 61, 65], Saxony-Anhalt (Germany) was situated in the former open-cast brown coal mine *Tagebau Goitzsche*, quarry field *Rösa-Sausedlitz*, east of Bitterfeld. Volunteer archaeologists discovered the site in 2002 [19] and the find layers were excavated thereafter by the *Landesamt für Denkmalpflege und Archäologie Sachsen-Anhalt—Landesmuseum für Vorgeschichte*. The sediments that contained the finds were silts and sands connected to a last glacial braided river terrace (Lower Terrace). Luminescence dating of the find layers yielded ages of 46.2±2.5 ka and 47.1±2.7 ka [19]. Unfortunately, the site was destroyed by a flood of the Mulde river. At the time of the excavation, the former mine was refilled to create a lake and the flood in 2002 raised the water level to the present state within a few days. However, the excavators recovered 371 artifacts, including seven refit sequences pointing to the relatedness of the find material [19, 51]. The 58 bifacial and unifacial tools are mostly characterized by a knife-like character, with sharp working edges often opposite a back, together with modified and unmodified pointed tips [1, 19, 51]. I included 3 bifacial and 3 unifacially shaped Keilmesser (Table 1) into the dataset, all of which are preserved in fresh condition.

Goitzsche (Saxony-Anhalt and Saxony), or Goitzsche Collection [19, 51], is an assemblage of late Middle Paleolithic artifacts that volunteer archaeologists collected between 1991 and 2002 in the same former open-cast mine where the site Pouch was located. All the finds stem from the basal layers of the last glacial river terrace deposits and were numerical dated to the onset of MIS 3, between 55 ka and 40 ka [1, 19]. I recently analyzed 1008 complete artifacts [19, 51] and the presence of prepared core blank production methods, together with the occurrence of Keilmesser, handaxes, bifacial and leaf-shaped scrapers attribute the assemblage to the late Middle Paleolithic of central and eastern Europe [8, 66–68]. I included 2 bifacial and one unifacially shaped Keilmesser into the present dataset. Except one which was affected by fluvial transport, the artifacts are preserved in good condition. This suggests that two of them have been collected from primary contexts. However, due to this preservational issues, I excluded the finds from most of the edge angle analyses.

The ongoing gravel pit Löbnitz (Saxony) [1, 51, 69, 70] is situated less than 1 km east of the former brown coal quarry *Tagebau Goitzsche*, quarry field *Rösa-Sausedlitz*. There, the gravels of the same Lower Terrace sequence as Goitzsche and Pouch are exploited by a floating dredger. Directly following the mining, the gravel was separated in different size fractions and the coarse gravel was dumped on a separate pile. From the latter, volunteer archaeologists and geologists have collected more than 3000 stone artifacts since the 1990s [69]. The sample of 838 complete artifacts that I analyzed recently [1, 51] includes Keilmesser, handaxes, leaf-shaped scrapers, and prepared core blank production methods. Therefore, and as there is no other gravel accumulation in this area other than the last glacial Lower Terrace sequence, it can be inferred that the artifacts collected in the gravel pit of Löbnitz originate roughly from the same chronological and geological context as the stone artifacts from the Goitzsche Collection and from Pouch. I incorporated 11 bifacial Keilmesser and 3 handaxes from the Löbnitz assemblage into the dataset. Although the shape of the tools is well preserved, the edges of the artifacts are preserved in varying conditions due to post-depositional processes like fluvial transport or mining. Therefore, I excluded the specimens from most of the edge angle analyses.

## Methods

### Technological characterization

Following procedures of technological lithic analyses [16, 48, 49], the technological characterization is based on five categories for the Keilmesser in the dataset: (1) the shaping of the surfaces, which includes also the roughing out the pre-form, (2) modifications of the back and the base, (3) the final modification and regularization of the working edge, (4) the modification of the distal posterior part, and (5) the thinning of the distal part and/or the tip. These categories provide the most important information about the manufacture and maintenance of these late Middle Paleolithic tools, e.g., the edge configurations and the distal thinning bear information about the strategies of edge angle maintenance [33]. I put my emphasis here on the analysis of the active edges, as they lie as well in the main focus of the subsequent 3DGM and edge angle analyses. I especially recorded the state of the distal posterior part as it was reported to play an important role as striking platform for thinning the distal volume of Central European Keilmesser [33].

### 3D geometric morphometric analysis and edge angles

I collected the landmarks on the 3D scans using *MeshLab* open-source software. Thereafter, I conducted the entire data processing in R [54]. Landmarks were processed using the package *geomorph* [52]. For further 3DGM analyses, like Procrustes superimposition, I applied algorithms of the package *morpho* [55]. To automatically measure the edge angles on the 3D scans, I used the package *Lithics3D* [53]. I created the diagrams of the present study with *ggplot2* [71].

#### 3D geometric morphometric analysis

3DGM is nowadays a widespread set of methods for quantitative analyses of stone artifact shape variability [1, 38, 45, 46, 72–78]. I applied it here with the intention to reveal patterns of variability within the Lichtenberg Keilmesser type and to further analyze the variability of the active edges.

The artifacts from Lichtenberg were 3D scanned using an ARTEC structured light scanner. The 3D dataset of Pouch, Löbnitz and Goitzsche was generated with a BIR Actis 225/300 CT-scanner with resolutions of 36 to 69 μm [1].

**Fig 3:**
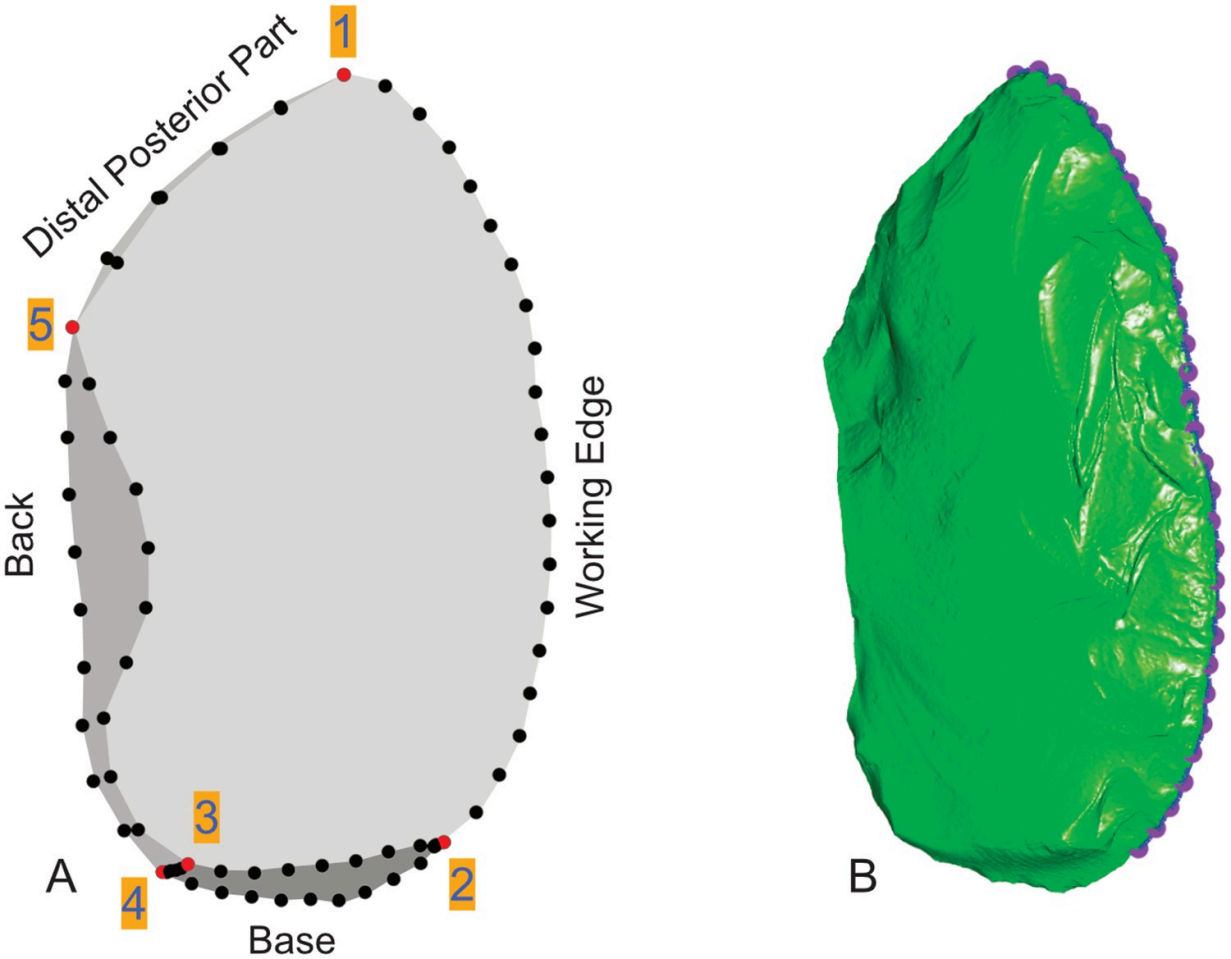
Landmarks and position of the edge angle measurements. A: 5 fixed landmarks: 1—tip, 2—proximal end of the working edge, 3—dorsal inflection point between base and back, 4—ventral inflection point between base and back, 5—inflection point between back and distal posterior part; 67 equidistant landmarks defining 8 curves: working edge, 2 curves at the base, the inflection between base and back, 2 curves for the back and 2 curves for the distal posterior part (if the distal posterior part is sharp they appear as one curve); B: automatically calculated path along the working edge with 30 equidistant landmarks between landmark 1 and 2 marking the positions of the edge angle calculations. Lichtenberg No. 54/54-11-35 (Landesmuseum Hannover).

For the 3DGM analyses, I collected 5 fixed landmarks (Fig 3A) at the following positions: the tip, the proximal end of the working edge, the dorsal and ventral inflection points between the base and the back, and the inflection point between the back and the distal posterior part. These points are present on all specimens in the dataset. Together with 67 semi-landmarks, equally spaced with the *geomorph* [52] package in R, they define 8 curves: the working edge, the dorsal and ventral outline of the base, the inflection between the base and the back, the dorsal and ventral outline of the back and the dorsal and ventral outline of the distal posterior part. The 3D outline shape is able to capture the morphology of the most important aspects (e.g., thickness, extension) of the tools’ individual techno-functional parts as well as the asymmetric shape of Keilmesser. Additional surface landmarks [73, 79] were not needed for the scope of the present analysis because it is not the surfaces but the edge configurations that define the Keilmesser-concept [12, 13].

I applied Procrustes superimposition to standardize orientation, scale and location of the landmark dataset [80]. In the next step, I analyzed the morphological variability in shape space with a principal component analyses (PCA) on the translated dataset of individual landmark configurations. I further conducted an additional cluster analysis on the principal component scores using the *kmeans* function in R with 1000 iterations and 10 random starts. The parameters of the function where adjusted until *kmeans* was stable and revealed the same clusters in every run. The application of the cluster analysis does not alter the patterns of the PCA result. It serves here mainly as a quantitative help to visualize clusters within the PCA result and to structure the interpretation. In a further step, I calculated the mean shapes of the individual clusters for comparison and to reveal patterns of morphological variability.

To analyze which parts are the most variable, I conducted the 3DGM analysis on two parts of the Keilmesser individually: (1) the working edge, and (2) the distal posterior part together with the back. I focused here on the active parts of the tools, as their morphologies are mostly affected and altered by retouch. Here, the distal posterior part is also interpretetd as an active edge as the distal part often creates a sharp working edge as well. Additionally, for the distal posterior part the curves of the back were included, as the morphology of the distal posterior part can only be understood in relation to the back. Important are, for example, the angle between the back and the distal posterior part or the length of the distal posterior part in relation to the extension of the back [51].

#### Edge angle measurements

The package *Lithics3D* by Pop [53] provides a function that automatically calculates edge angles from 3D models at equidistant fixed points along an edge. For my study, I chose 30 equidistant points and measured the angle at 5 mm from the edge (Fig 3B). As the back and the base are thick, often naturally prehensile parts by definition, I focussed on the edge angles of the active parts, the distal posterior part and the working edge.

The *edgeAngles* algorithm computes the angles along a path defined by ordered surface coordinates, at a given distance (here: 5 mm) perpendicular to the path. This function works by first computing planes perpendicular to the edge by implementing the *curve.pp* function. Once these planes have been obtained, mesh edges that intersect the planes are identified with the *edgesOnPlane* algorithm. In a subsequent step, *edgeAngles* uses the *e2sIntersect* function to compute the intersection points of these mesh edges with a sphere of radius in mm to identify the location where the mesh thickness should be measured. The intersections with the greatest distances between them are then used to measure mesh thickness, and angles are then computed using simple trigonometry [53]. There was only one case from Lichtenberg (54/45-8-64) where the edge angles could not be measured. This artifact was refit from two fragments (transversally broken) and because of a small gap on the working edge, the algorithm for the automatic measurement failed.

In addition to the edge angle analysis, algorithms of the package *Lithics3D* were applied to automatically measure the maximum length, width and thickness of each artifact.

## Results and preliminary discussion

### Technological description

**Fig 4:**
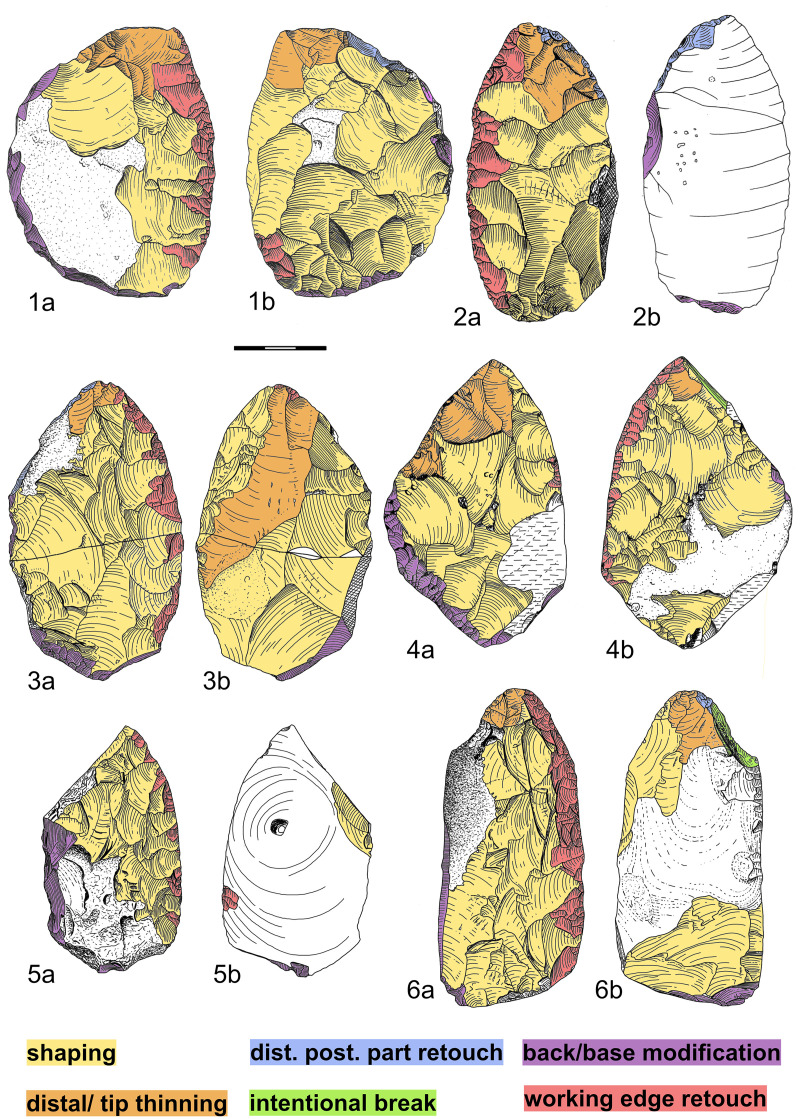
Main technological features and technological variability of Lichtenberg Keilmesser. 1-2 Pouch, Saxony-Anhalt (Landesamt für Denkmalpflege und Archäologie Sachsen-Anhalt), 3-6 Lichtenberg, Lower Saxony (Landesmuseum Hannover). 1: Keilmesser with rounded outline shape and retouched distal posterior part as striking platform (2004:8679,55); 2: unifacially shaped Keilmesser with retouched distal posterior part as striking platform (2004:8679,6); 3: Keilmesser with slightly retouched distal posterior part as striking platform (49/48-3-24); 4: Keilmesser with distal posterior part as a striking platform established by an intentional break. The break is considered as intentional, as it cuts older flake scars and was used as striking platform for subsequent retouch (51/52-3-1); 5: unifacially shaped Keilmesser made on a frost shard, unworked distal posterior part, no tip thinning (50/48-4-23); 6: Keilmesser with distal posterior part as a striking platform established by an intentional break. The break is considered as intentional, as it cuts older flake scars and was used as striking platform for subsequent retouch (48/49-2-13). Drawings and Graphic: M. Weiss, 4 redrawn after Veil et al. 1994.

Fig 4 shows examples of the technological variability of Lichtenberg Keilmesser. Special characteristics of the distal posterior part are summarized in Table 2.

**Table 2:** Technological characteristcs of the distal posterior part and the tip region.

Site	Edge (increasing angles towards the back)	Edge—fine Striking Platform	natural Surface/Cortex	intentional Break	steeply angled and/or very round distal posterior part: present/not present	thinned Tip: present/not present
Lichtenberg	13	2	3	4	2/20	20/2
Pouch	4	2	0	0	1/5	6/0
Löbnitz	11	0	0	0	2/9	11/0
Goitzsche	1	1	1	0	1/2	2/1

Surface shaping, which the knappers carried out in a bifacial and unifacial way [1], was done directly from the working edge, the base, and from the back (Fig 4). The latter is common in LMP assemblages from Central Europe, as suggested by Iovita [33] and demonstrated by refits from Pietraszyn 49a [22]. Shaping from the back, often carried out on the flat ventral side, thins the entire tool volume and is applied in the initial stages of tool manufacture but also as resharpening solution when the piece gets proportionately thicker [33]. More detailed examples are given in the section about edge angles and Keilmesser reduction below. Prior to the final edge regularization (Fig 4) the surface along the lateral working edge was thinned more precisely with removals directly from the working edge, equivalent to the second non-KMTB (re)sharpening solution after Iovita [33].

The distal posterior part is often angled towards the working edge to form an often rounded, a more or less pointed tip with the latter. Only in a few cases (Table 2; Fig 2:3) the distal posterior part is rather steeply angled and/or very round with no pointed tip. The thinning of the distal volume, i.e. the tip, is a very common feature across all assemblages of the dataset (Table 2). This thinning was mostly realized from the distal posterior part. Either it was done perpendicular to the working edge from the middle and proximal part of the distal posterior part (Fig 4:1a, 4:2a, 4:3a, 4:4a and 4:6a) and/or parallel to the working edge and struck from the distal edge of the distal posterior part (Fig 4:1b, 4:2a, 4:3b, 4:4a and 4:6b). One Keilmesser from Pouch (Fig 4:1b) shows a removal directly along the working edge that may represent a former tranchet blow. However, this cannot be proven as it is only partly preserved and it therefore belongs to an earlier stage of distal thinning before the tool was potentially resharpened. The latter is also evidenced by a neighbouring highly reduced shaping scar along the same edge (Fig 4:1b). Fig 4:3 illustrates that removals from the distal posterior part could also thin out the entire piece. Neanderthals designed the distal posterior part as striking platform for these surface removals. This was either achieved by coarse or fine preparation, a thick edge, a thick natural surface, or an intentional break (Fig 4:6b). The latter was only observed in Lichtenberg.

The base and the back consist mostly of natural surfaces or bear some modifications by coarse retouch. The base is often unworked. However, in some specimens the base was retouched as a striking platform for shaping (Fig 4:2) or was modified by non-invasive retouch on the surfaces (Fig 4:4).

The lateral working edge is predominantly convex, although there is some variation. I will come back to this in the section about the 3DGM results below.

### 3DGM

**Fig 5:**
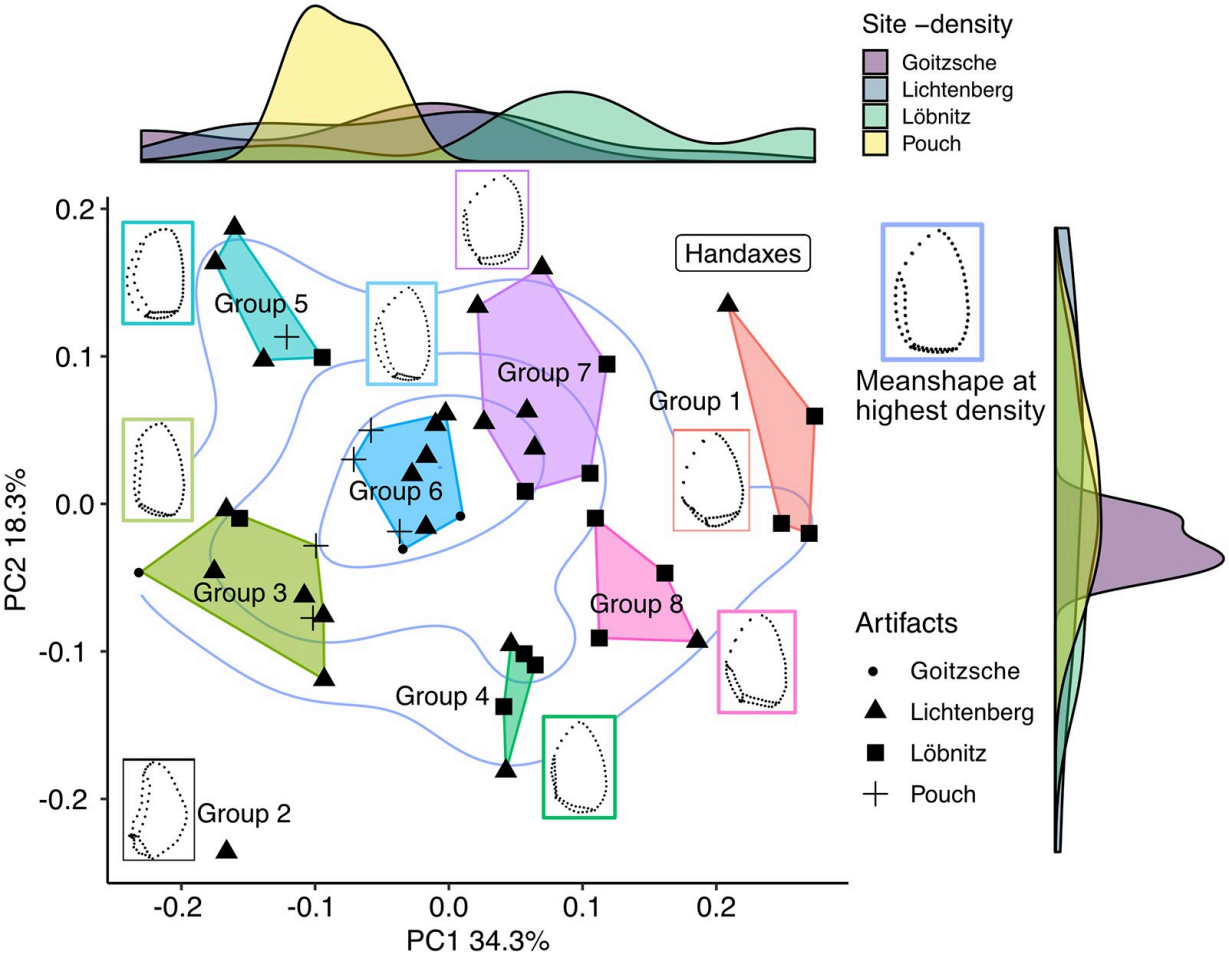
The first two principal components in shape space of 46 bifacial and unifacially shaped tools. Lichtenberg: 19 bifacial Keilmesser, 3 unifacially shaped Keilmesser, 1 handaxe; central Germany (Löbnitz, Pouch and Goitzsche): 16 bifacial Keilmesser, 4 unifacially shaped Keilmesser, 3 handaxes. Clusters were automatically generated using the kmeans function in R with 1000 iterations and 10 random starts. The specimens with dotted lines represent the mean shapes of each group. Isolines show the density areas for all tools, the red line marks the highest density. The mean shape for the highest density is given in the upper right corner of the graph. Density graphs at the plot margins show the site density for each axis.

Fig 5 displays the first two principal components in shape space of 46 bifacial and unifacially shaped tools. The center of the entire plotting area has the highest density of tools. Furthermore, the density graphs at the plot margins indicate that the tools from Lichtenberg scatter over the entire plot area and overlap with the tools from central Germany. With regard to the limiting factor of low sample size for the distribution of artifacts from Pouch and Goitzsche, this overlap suggests a strong relatedness of the tool designs from both Lichtenberg and the central German assemblages Pouch, Löbnitz, and Goitzsche (Fig 1). However, there are some differences within the central German dataset. The artifacts from Goitzsche and Pouch scatter on the left half of the PC1 axis, whereas the Löbnitz tools are distributed more two the right. But regarding PC2 they are all, including Lichtenberg, centered on the axis.

The visual inspection of the result (Fig 5) reveals that principal component 1 displays an increase of broadness and in the oval outline shape of the tools, as well as an increase in the elongation of the distal posterior part. More narrow Keilmesser plot on the left extreme of PC1, whereas the more symmetric, broad and oval handaxes plot on the right side. PC2 separates the tools regarding the morphology of their base and the back. The position of specimens with broader backs and/or bases increases with PC2 (for similar results see [1]).

**Fig 6:**
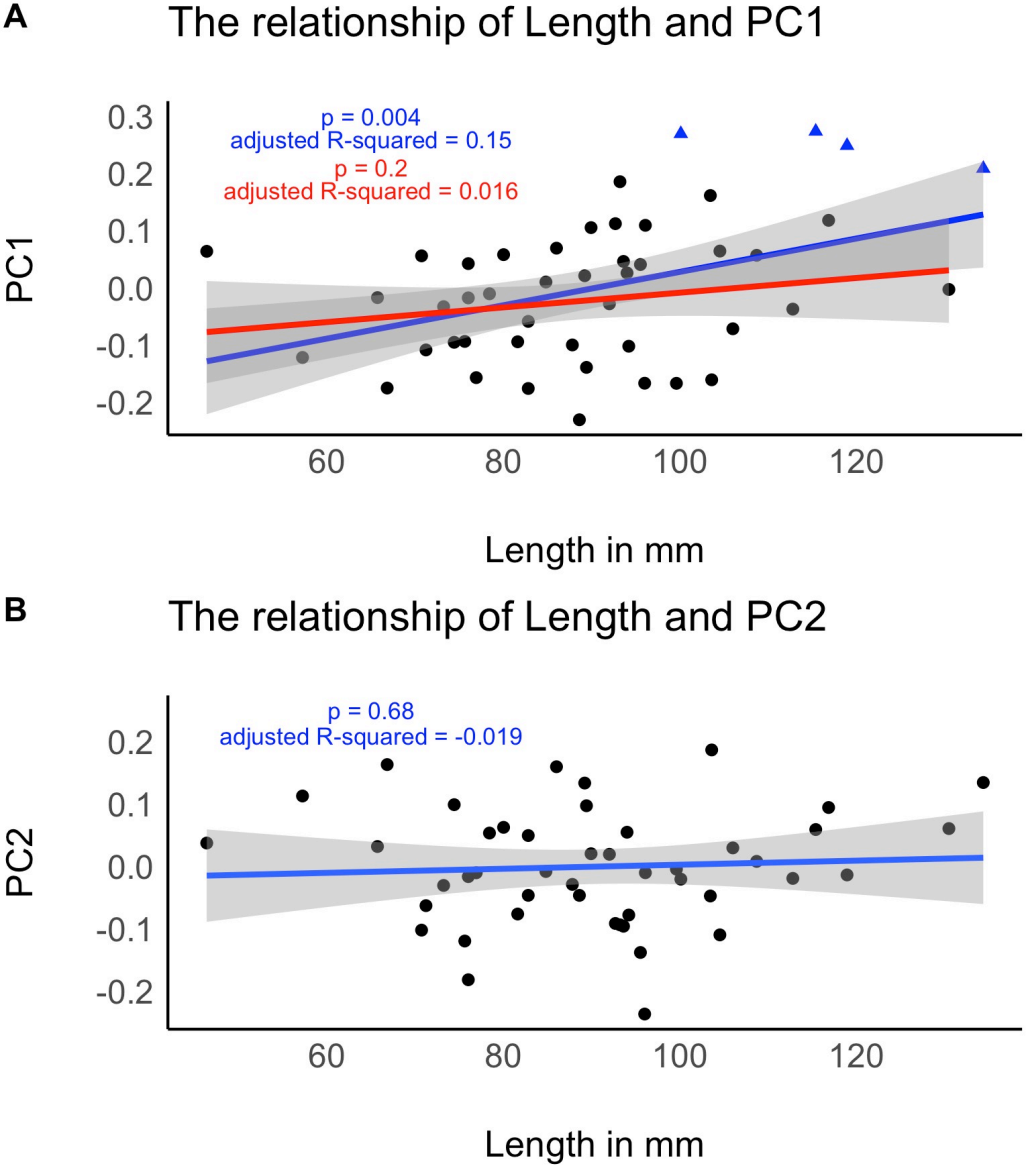
The relationship of size and the two main principal components. A: The relationship of Length and PC1. Blue color refers to the inclusion of handaxes, red means handaxes are excluded from the regression. Blue triangles mark the position of handaxes. B: The relationship of Length and PC2.

I also inspected if the shape variation that we see in Fig 5 is dependent on size (Fig 6). I used the length of the tools as a representation of size, as elongation is a main feature of bifacial backed knives (see Figs 2 and 4). At a significance level of p = 0.05, length and PC1 have a significant relationship. To evaluate if this is also the case for Keilmesser only, I excluded the handaxes from this analysis. We already saw that they form a separate morphological cluster at the upper extreme of PC1 (Fig 5), separating them from Keilmesser. Without the handaxes, the relationship is not significant anymore (Fig 6A). In other words, the morphological variation of Keilmesser is independent of size, and—as last consequence—also of a decrease in size during reduction. This is reinforced by the result for length in relation to PC2, as there exists also no significant relationship (Fig 6B). On the other hand, the separation of handaxes from Keilmesser is not only due to shape, but depends also on handaxes being different in size.

Due to the low sample size of handaxes, the robustness of the result (Fig 6A) for the relationship of size and PC1 needs to be inspected further. Therefore, I resampled the data 1000 times with replacement and excluded 10 specimens in each run. The results are listed in Table 3 and demonstrate that when handaxes are included the relationship of size and PC1 is significant in 75% of the cases. In contrast, assemblages without handaxes give a significant result for the relationship of size and PC1 in only 19% of the cases. These results confirm the initial observation that the shape variability of Keilmesser is mostly independent of size.

**Table 3:** Resampling of the data 1000 times with replacement and the exclusion of 10 specimens in each run.

Handaxes	Significant	n	Mean_p	Percent
not present	no	34	0.38	81
not present	yes	8	0.02	19
present	no	235	0.18	25
present	yes	723	0.01	75

The table shows that when handaxes are included, 75 percent of the relationships of PC1 and size are significant. If handaxes are excluded, around 81 percent of the relationships are not significant. This indicates that Keilmesser shape variability is mostly independent of size. Note that the resutlt could change slightly, every time this document is created.

The cluster analysis on the principal component scores revealed 8 different groups. Thereby, all the groups incorporate tools from at least two sites. This suggests that the variability within the dataset is not structured by site. Of course, this result is only tentative, as some sites have smaller sample sizes than others. Further, it has to be kept in mind that these groups are mainly a help here to interpret the patterns of the PCA result and to visually structure the shape variability. The groups are no fixed “natural” clusters and rely a lot on the parameters of the *kmeans* function. In other words, the groups do not represent sub-types. Further, the groups do not alter the result of the PCA, i.e., specimens that plot closer together nevertheless share more shape similarities than tools that plot further from each other. Additionally, it is important to keep in mind that PC1 and PC2 represent a shape continuum where the tool morphologies form rather gradients. Therefore, an alternative way of inspecting the morphological relatedness is presented below. I interpret the variability in the clusters tentatively based on their mean shapes as follows:

1. Group 1 consist exclusively of handaxes. They differ from Keilmesser in having a symmetrical tip formed by two lateral working edges. One of the working edges is slightly shorter and connected to a short back, and is interpreted here as equivalent to the distal posterior part of Keilmesser. Similar to the results of my previous study [1], the handaxes form a rather tight group within the plot. This was an expected result and suggests that the 3DGM is in fact measuring aspects of edge variability of interest here. The morphological separation of handaxes from Keilmesser is reinforced by the fact that they plot outside the highest density areas of the scatter plot (Fig 5).
2. Group 2 is represented by a single specimen that represents an outlier within the dataset. Although it consists of the morphological parts defined for Keilmesser, the back is elongated and concave, the distal posterior part is very short, and the working edge is extremely convex.
3. The Keilmesser of Group 3 are rather elongated, with a short and steeply angled distal posterior part and a straight back.
4. Group 4 forms a rather tight cluster. The mean shape points to an oval shape with a symmetric tip. This symmetrical tip is typical for handaxes, but the distal posterior part is here rather short compared to handaxes. The base and the back are relatively thin in Group 4. In this group falls one of the Keilmesser that Veil [8, 14] would classify as Faustkeilblatt (Fig 2:7).
5. Group 5 comprises specimens with a tendency to have an overall elongated shape, a long, broad and convex back, and a short and steeply angled distal posterior part.
6. Group 6 forms the major part of the highest density scatter in the plot. The meanshape of the Keilmesser tends to have a narrow base, a broad back and a longer distal posterior part than Groups 2, 3 and 5. Thereby, the distal posterior part is straight instead of slightly convex and together with the working edge it forms a more pointed tip.
7. The distal posterior part of Group 7 is similarly straight as in Group 6, forming also a pointed tip. But in contrast, the back is here more narrow and the base longer. However, Groups 6 and 7 are fairly similar.
8. The mean shape of Group 8 is characterized by an elongated distal posterior part, a rounded tip, and a relatively short back which is angled towards the proximal side of the Keilmesser. With this morphological features, the group is close to handaxes, reinforced by its position in the plot closest to the latter. In the Group 8 also includes one of the Keilmesser that Veil [8, 14] would classify as Faustkeilblatt (Fig 2:5).

Despite these groups I also calculated the mean shape for the highest density of the scatterplot. This serves here as an additional measure for morphological relatedness independent from the cluster analysis. 13 out of 46 tools are concetrated in this area and represent Keilmesser with the closest shape relation within the dataset. The highest density area is represented by Group 6 and the lower left part of Group 7. Further, the group is dominated by Keilmesser from Lichtenberg and Pouch, but one specimen from Löbnitz and two out of three artifacts from Goitzsche are present as well. The mean shape of the highest density area resembles the definition that Veil [8] gave for the “ideal” Lichtenberg Keilmesser: an oval outline shape with a longitudinal symmetry, especially in the tip region, a convex working edge that forms a retouched tip at the distal part, and a relatively long back. In the light of the data presented here up to now, this may point to the presence of an underlying form-function template for these tools.

The 3DGM result does not clearly confirm the separation of the tools from Lichtenberg into Keilmesser and Faustkeilblätter as suggested by Veil [8]. Despite that handaxes form a tight cluster outside the highest density areas and are generally larger, there seems to exist a shape continuum along PC2: from elongated pieces with a long back and short distal posterior part on the left, to broader pieces with longer distal posterior parts and more symmetrical tips (Faustkeilblätter) at the center right, thorough to handaxes with symmetrical tips, oval shapes, long distal posterior parts and short backs on the right. Faustkeilblätter also plot together in groups with slightly different Keilmesser variants. This rather confirms the similar interpretation of a shape continuum between these tools by Jöris [13].

**Fig 7:**
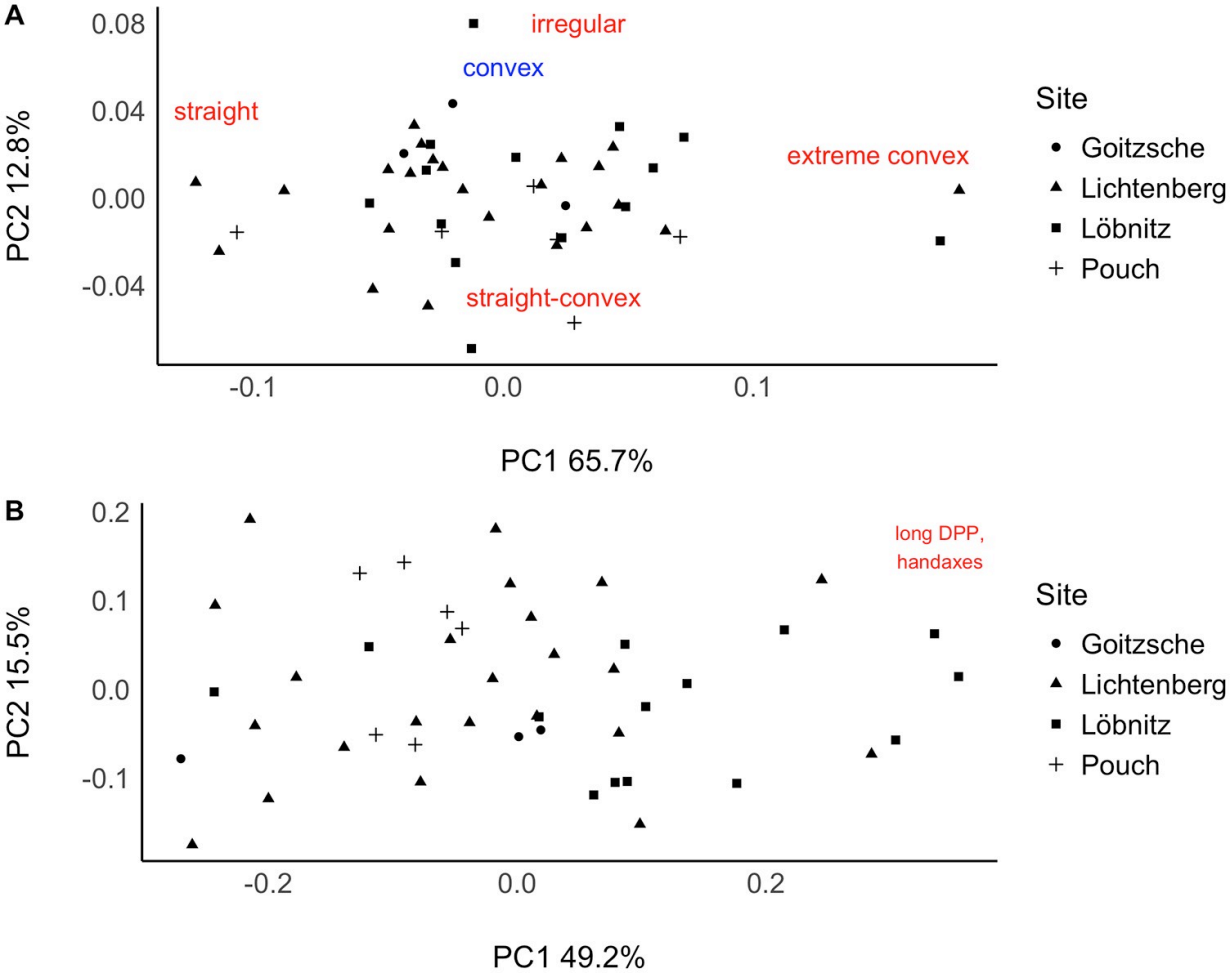
The first two principal components in shape space of A: The cutting edge and B: The distal posterior part and the back. A: Tools with convex cutting edges cluster in the center of the plot. The Categories: convex, straight, straight-convex, extreme convex, irregular represent tendencies. B: Only tools with a long distal posterior part (DPP), handaxes and Faustkilblätter, are separated at the right plot margin. The other tools spread over the entire plotting area suggesting a high degree of variability.

Despite morphological differences, the convex working edge is common to the mean shapes of all groups. The most variable parts instead seem to be the distal posterior part, the base, and back. To inspect this observation further, I conducted the 3DGM analysis on the back and the distal posterior part on the one hand, and the working edge on the other hand individually (Fig 7).

The working edge indicates a low variability, as most of the tools with a convex working edge shape are concentrated in the center area of the plot (Fig 7A). Thereby, more straight working edges tend to plot towards the left part of the PC1 axis, whereas straight-convex edges are situated more in the lower part of the PC2 axis. One specimen from Löbnitz has an irregular edge shape and is separated in the upper extreme of PC2. Two tools with extreme convex working edges plot outside the main cluster at the upper extreme of PC1. One of them is again the specimen of the outlier Group 2 within the main PCA (Fig 5).

Figure (Fig 7B) shows the result for the first two principal components in shape space of the distal posterior part and the back. In contrast to the result for the working edge, the shapes of the distal posterior parts and the backs form no clusters and scatter over the entire plotting area. Only the handaxes, with their elongated distal posterior part are separated to the right along PC1, confirming their different distal posterior part morphologies compared to Keilmesser. The result suggests that the highest variability of Lichtenberg Keilmesser is concentrated indeed in the shape of the distal posterior part and its relation (length, angle) to the natural morphology of the back. The latter, of course, creates also a high degree of variability.

I could show that the working edge is relatively constant in its convex shape, confirming the observations made by Veil [8, 14] and Jöris [13] for the Lichtenberg Keilmesser. In contrast, the second active edge, the distal posterior part, is in its morphology a highly variable part of the tool. According to the hypothesis by Iovita [33] this part (and parts of the back) is used as a striking platform for thinning the distal volume on the tools. This is an observation that is indeed common in the dataset (Fig 4, Table 2). And subsequent thinning may, in my opinion, alter the length and shape of the distal posterior part, causing variability. In contrast, Veil [8] defines the distal posterior part as a fixed extension of the working edge around the tip that forms a second sharp edge. To evaluate these two contrasting views, I am going to present the results for the edge angles of the distal posterior part and the working edge in the next section.

### Edge angles

**Fig 8:**
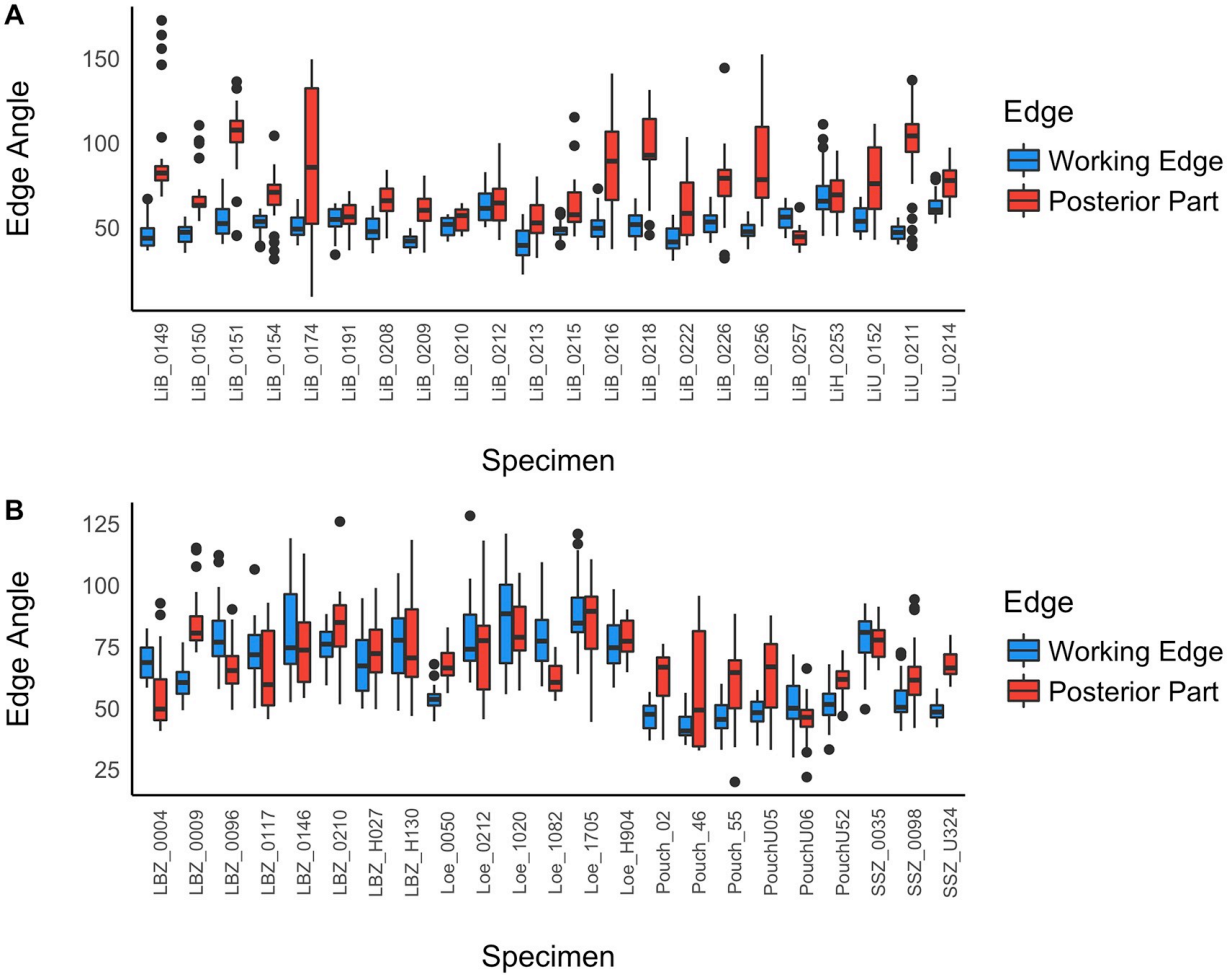
Comparison of the edge angles of the working edge and the distal posterior part. A: Lichtenberg. LiB = bifacial tool, LiU = unifacially shaped tool, LiH = handaxe; B: central Germany. No letter before the number: bifacial tools, U before the number: unfacially shaped tool, H before the number: handaxe. Note the difference between the assemblage of Löbnitz, where the edges are potentially affected by fluvial transport and the edge angles of the excavated assemblage of Pouch and some in situ finds from Goitzsche.

The boxplots in Fig 8 compare the edge angles of the distal posterior part for the tools from Lichtenberg and from central Germany. The edge angles for Keilmesser are generally larger on the distal posterior parts than on the working edges, whereas for handaxes the angle ranges mostly overlap. The latter was expected, as handaxes are defined as having two working edges. An exception for Keilmesser is Löbnitz, where the edge angles are generally higher compared to the other assemblages. Furthermore, the range of the angles for the distal posterior parts and the working edges overlap more often in Löbnitz than in the other assemblages. This confirms the observation stated earlier that the edge angles of Löbnitz have to be regarded with caution, as there is a high potential for post-depostional edge damage in this assemblage. Similar observations are true for the Goitzsche specimens. Therefore, both assemblages will be excluded from the following edge angle analyses and I am going to work only with the excavated assemblages Lichtenberg and Pouch. The remaining handaxe from Lichtenberg will be excluded for its sample size of 1. From now on, I will especially focus on the Keilmesser as main subject of the study.

The angles of the distal posterior part for the Keilmesser of Lichtenberg and Pouch are centered between 57.5° and 78.5°:

Min. 1st Qu. Median  Mean 3rd Qu. Max.
44.00 57.50 66.00  69.52 78.50 108.00

As already shown in Fig 8, the angles of the working edge are centered lower:

Min. 1st Qu. Median  Mean 3rd Qu. Max.
39.00 47.00 49.00  49.19 52.50 61.00

The larger edge angles for the distal posterior part on Keilmesser suggest a different function of this active edge compared to the working edge with its relatively lower angles. The larger angles point towards a function such as striking platform and/or an extension of the prehensile part of the tool. However, Veil [8] and Jöris [13] observed a second sharp edge in the distal part of the distal posterior part on the Lichtenberg tools. To evaluate this observation, we need to look at the distribution of the edge angles along the active edges of Keilmesser.

**Fig 9:**
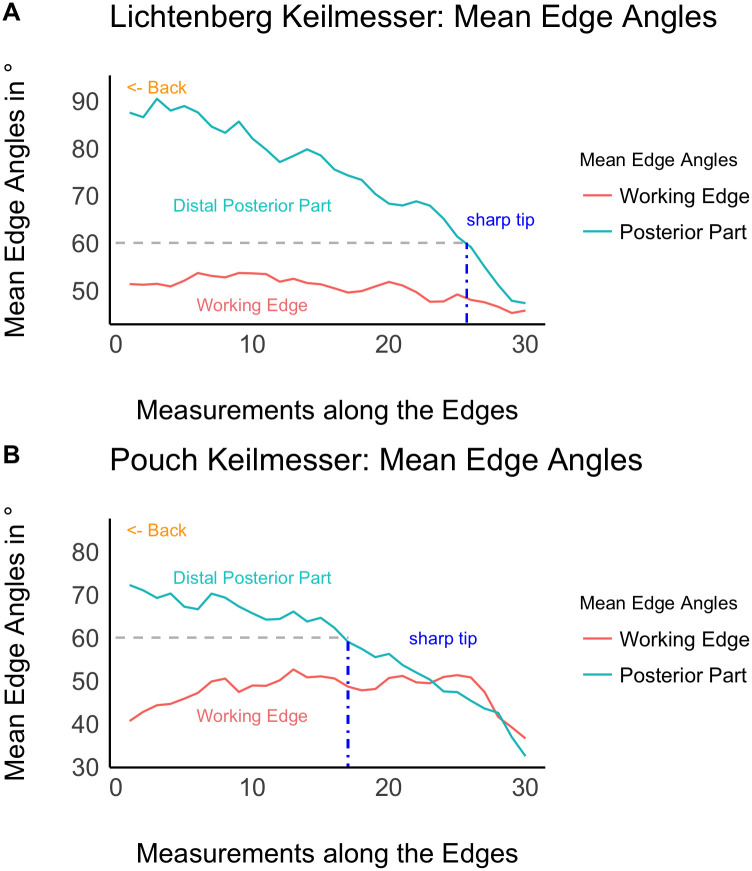
Mean edge angles of the distal posterior part and the working edge. The mesurements extend from the end of the back (left) to the tip (right). A: Keilmesser from Lichtenberg; B: Keilmesser from Pouch. Because of preservation issues, only the Keilmesser from the excavated assemblage Pouch are included here.

The mean edge angle graphs for Keilmesser from Lichtenberg and Pouch in Fig 9 can be viewed as edge morphology translated into angles. As plotted here in actual edge direction, the edge angle values almost perfectly resemble visually the distal morphology of Keilmesser. In other words, there is a high potential that edge angles and edge angle management on the active edges have an influence on tool morphology. And this pattern is not only visisble in Lichtenberg, but also in the assemblage of Pouch (however, note that in Pouch the distal posterior part can also be thinner than the distal part of the working edge). That means that this morpho-technological principle was applied in different regions of the northern central European Plain.

The edge angles are distributed differently on the two active edges (Fig 9). To draw inferences of differing edge functionality, a morpho-functional threshold is set here at 60°, because acute edge angles <60° are interpreted as sharp and suitable for cutting tasks [81]. The working edge has a relatively even distribution of angles, suggesting constant edge functionality along the entire edge. Thereby, the mean angles are distributed around 49°, which means a sharp edge suitable for cutting tasks. In contrast, the angles of the distal posterior part decrease towards the tip. With the mentionned threshold at 60°, I can divide the distal posterior part into two morpho-functional parts: (1) approximately two thirds of the edge have large angles, extending the back as prehensile part on the one hand, and serving as a striking platform (natural and/or retouched, see Fig 4 and Table 2) for distal thinning on the other hand. (2) The distal third of the distal posterior part is below 60° and constitutes a sharp tip together with the distal part of the working edge. From a functional point of view this sharp tip seems to have been important for certain cutting tasks. A slight difference between the assemblages is that the sharp part is shorter in Lichtenberg than in Pouch. Both morpho-functional parts suggest the distal posterior part as a multifunctional edge for prehensile aspects, distal thinning, and potentially cutting.

### Edge angles and keilmesser reduction

In the final part of this study, I combine the results from the technological, 3DGM and the edge angle analysis. I focus here on the analysis of the working edge as main active edge. Thereby, I assume that high edge angles are an indication for a decrease of edge functionality. In the beginning, my aim is to inspect if the grouped morphologies of Keilmesser are related to subsequent reduction/resharpening. Or, in other words, if the mean shapes for the groups are influenced by resharpening. I already showed above (Fig 6) that shape variation of Keilmesser is independent of size and now I want to analyze this aspect further in looking at the edge angles. Because of issues with edge angle preservation in collected assemblages, I include here only on the excavated artifacts.

**Fig 10:**
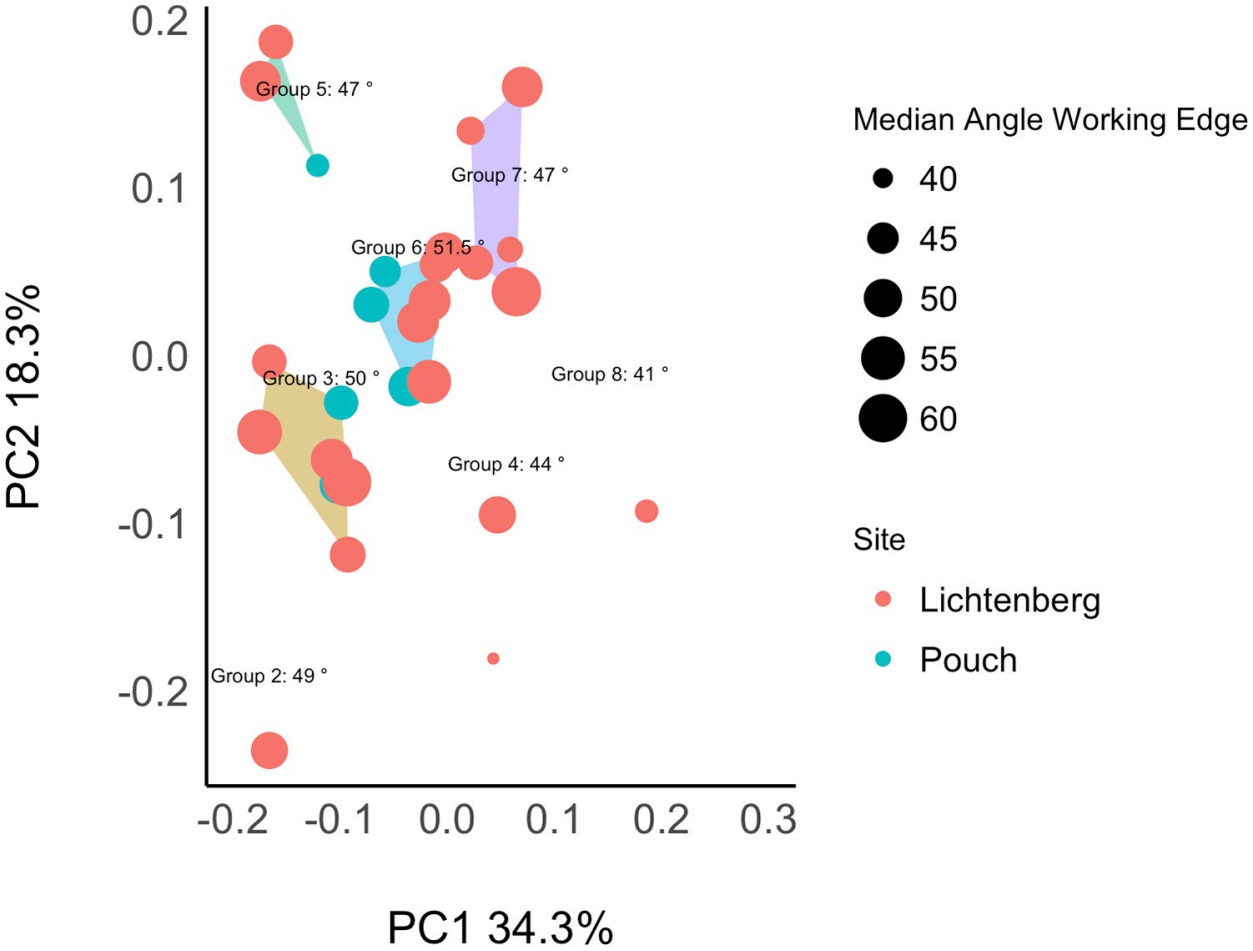
Groups of the first two principal components in shape space (Keilmesser only) combined with the median working edge angles of each tool. The median edge angles for each group are also displayed. Because of preservation issues, only the Keilmesser from the excavated assemblages Pouch and Lichtenberg are included here. Groups with less than two artifacts are displayed here, but excluded from further interpretations discussed in the main text.

Fig 10 shows the groups from the 3DGM results together with the median working edge angle for each tool and for each group. The median group angles with values between 47° and 51.5° (for groups with more than two artifacts) indicate that no group of more than two artifacts consists exclusively of more or less reduced pieces, respectively. It is obvious that the tools with their differing median angles are distributed evenly among the groups. This is further underpinned by the result for a one-way ANOVA:

   Kruskal-Wallis rank sum test
data: Median_Angle_WE by Group
Kruskal-Wallis chi-squared = 2.5066, df = 3, p-value = 0.4741

At a 0.05 significance level, I conclude that the median working edge angles per specimen of the groups with more than two artifacts are identical populations. In other words, there is nor relation between shape change and working edge angles of Keilmesser in the dataset. The angles of the working edge are relatively constant compared to overall shape. Viewed from upside down, this also implicates that the specific morphological characteristics of Keilmesser can be found on tools with differing working edge angles.

But does that mean that there was no heavy reduction or resharpening in both assemblages? To inspect this, let’s take a look at the reduction pattern in the dataset. As a measure for reduction, I use the relative thickness index (RTI) [61, 82], calculated as:

$$\sqrt{maxLength*maxWidth}/maxThickness$$

This index represents the thickness of each artifact in relation to its surface area. If the value of this measure decreases, the artifact gets thicker in relation to its surface area and may indicate an increase in overall reduction. However, there is also some caution needed in the interpretation of the RTI, as artifacts manufactured on blanks with a naturally thick back can also have high maximum thickness values.

The RTI of the Keilmesser from Lichtenberg and Pouch is distributed as follows:

Min. 1st Qu. Median  Mean 3rd Qu. Max.
2.840 3.485 3.897  4.009 4.381 6.740

The summary statistic shows that the data is not normally distributed. This is related to an outlier, a Keilmesser or Faustkeilblatt (Fig 2:7) which was manufactured on a very thin blank. If this outlier is removed, the values are more normally distributed:

Min. 1st Qu. Median  Mean 3rd Qu. Max.
2.840 3.460 3.793  3.904 4.331 5.232

**Fig 11:**
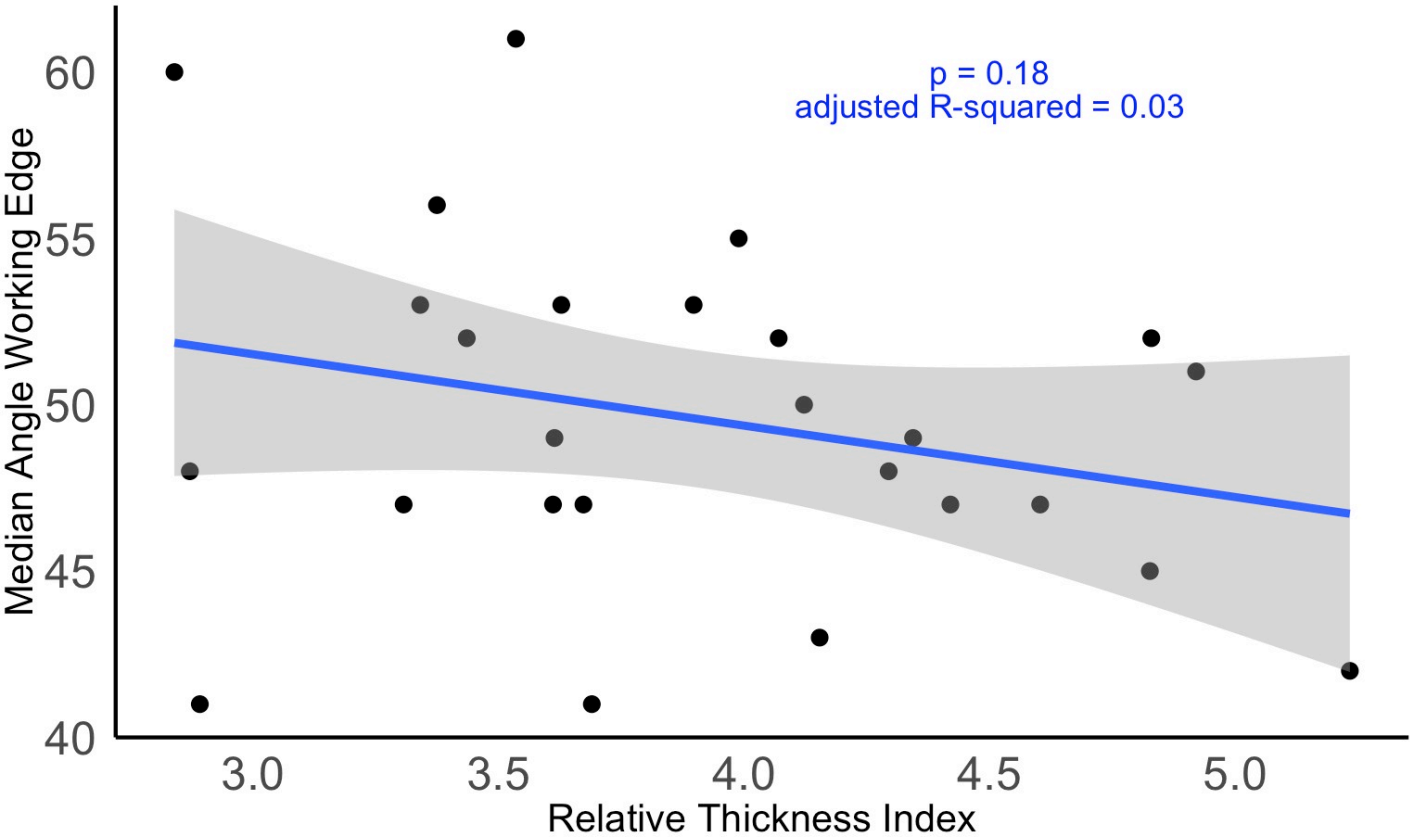
Relative thickness in relation to the median working edge angles on Keilmesser from Lichtenberg and Pouch. This graph shows that there is no significant relationship between RTI and median working edge angle when the outlier is removed.

The data shows that the RTIs for the Keilmesser are distributed in a relatively narrow range, most of the specimens have values between 3.46 and 4.3. But what does this mean for our dataset? Let’s inspect the relationship between the measure of reduction and the median edge angle on the working edges. If the outlier is included, we can see a significant relationship between the two variables at a significance level of 0.05 (p = 0.02). However, the low adjusted R-squared (0.154) suggests a high variation of the data and suggests a rather weak relationship. If the outlier of the exceptionally thin Keilmesser is removed (Fig 11), the relationship looses its significance. In other words, when the artifacts get thicker, either through edge modification in the framework of the initial manufacture, or resharpening, the edge angles were kept rather constant. Based on the data presented here, I infer that Neanderthals had ways to keep the edge angle low and preserve the functionality of the working edge during manufacture, use and subsequent reduction.

**Fig 12:**
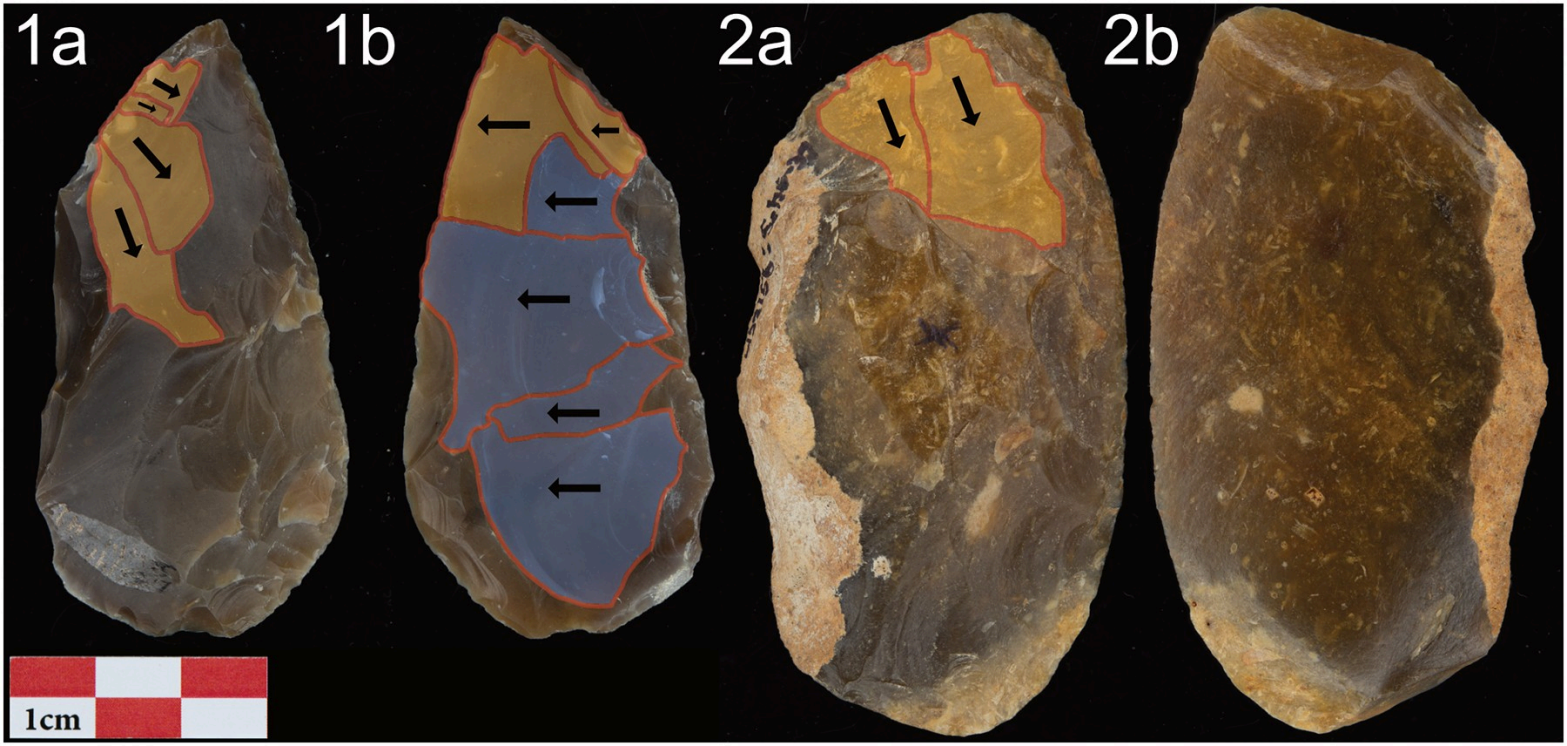
Two examples of reduced Keilmesser from Lichtenberg. Yellow scars: thinning from the DPP; Blue scars: thinning from the back. 1: Keilmesser (56/47-14-42) with dorsal tip thinning form the DPP and thinning of the entire ventral face from the back. Despite heavy reduction an acute median angle (53°) of the working edge is maintained; 2: unifacially shaped Keilmesser (53/56-6-39) with dorsal tip thinning. The two scars hinged within the thick volume in the center of the piece and could not thin the volume entirely. The median working edge angle of 60° could not be reduced further.

This is illustrated by the values for the angles of the working edges. As we already saw in Figs 8 and 10, the angles of the working edges of Keilmesser from Lichtenberg and Pouch are mainly below 60° with a median of 49°. There are only two artifacts with a median angle of 60° (example 53/56-6-39 below) and an angle of 61° (Fig 2:6), respectively. And even those pieces are just hitting the defined value of <60° for cutting purposes. In the following, I picked two examples from Lichtenberg, to illustrate strategies to maintain the edge angle, one successful (Fig 12:1) and one not (Fig 12:2).

Keilmesser 56/47-14-42 (Fig 12:1) looks technologically heavily reduced compared to the other artifacts: it is relatively small and narrow, and the flake scars on the dorsal face suggest that the use-life of the piece started with a larger size. However, the median working edge angle of 53° degrees would not suggest a heavily resharpend tool. But a closer look at the artifact reveals that Neanderthals took care repeatedly to maintain the angle of the working edge to preserve its functionality. As observed by Iovita [33], they thinned the volume from the distal posterior part, but here alternating on the dorsal and ventral side. As the Keilmesser got thicker and narrower, they reduced the volume of the entire ventral face using also the back as a striking platform. This increased the RTI to a value of 3.9 (the mean of the dataset) and the angle of the working edge could be kept low. In the distal half of the tool, the removals from the back and the distal posterior part were extensive enough to thin the entire surface inlcuing the working edge. The latter was subsequently regularized by dorsal retouch. In contrast, on the proximal half of the tool thinning removals from the back did not reach the working edge. Here, the knappers changed their strategy. Through ventral edge retouch, they created a striking platform for subsequent dorsal thinning removals perpendicular to the working edge.

In contrast, the resharpening of the unifacially shaped Keilmesser 53/56-6-39 (Fig 12:2) seems to have been given up when the working edge angle became too steep (median 60°). Also, it has one of the lowest RTIs with a value of 2.8. The knappers tried to thin out the distal volume from the distal posterior part with two large removals. But the two removals in the thick center of the piece hinged. Further dorsal thinning from the distal posterior part was therefore not possible any more. The striking platform of the distal posterior part was oriented to remove flakes from the dorsal face, and this also prohibited the thinning of the ventral face as an alternative solution. The thick cortical and irregular back also provided no suitable angles for surface removals.

These two examples and the results of the reduction, edge angle and 3DGM analyses show three things: firstly (1) Lichtenberg Keilmesser seem to have had a long use life and there functionality was maintained as long as possible, (2) the tool may potentially have been ultimately discarded when the angle increased above 60° and the edge lost its functionality (cutting), and (3) edge angle maintenance is independent of morphological groups and the individual techno-functional units, and angles where preserved during resharpening.

## Discussion

I could show in the present analysis that: (1) Lichtenberg Keilmesser form morphological sub-groups in the PCA result that are, keeping in mind sample size, not structured by the sites within the dataset, (2) Keilmesser shape and shape change is independent of size, (3) there is a shape continuum at least between Keilmesser and Faustkeilblätter, (4) handaxes form a tight cluster outside the density areas for Keilmesser due to their symmetric morphology and difference in size, (5) the convex lateral working edges are rather standardized in angle and shape, (6) the distal posterior part in relation to the back is the most variable active part in its morphology and angle, and represents a multi-functional edge, (7) edge angle distribution along the active edges influences edge shape, (8) the establishment and maintenance of a sharp and thin distal tip seems to be an important feature of these tools, (9) edge angle maintenance is not related to overall shape alteration, and (10) tools may have been ultimately discarded when the functionality of the edges (i.e. the angles) could not be maintained. In the following, I will discuss some of these aspects further.

### Form-function and angles

The 3DGM analysis has shown that the Lichtenberg Keilmesser has an inherent morphological variability across assemblages which resulted in specific, although artificial, sub-groups. This confirms similar earlier observations by Jöris [13] and Veil [8]. Thereby, the presence and positions of the individual techno-functional units stay constant. Furthermore, these units are independent of size, which was already suggested by Iovita [37] and confirmed by Frick et al. [30, 32]. The overall shape varies between more elongated and broader specimens, from short and angled distal posterior parts to elongated distal posterior parts with a symmetric tip area. The latter is represented by Faustkeilblätter, which lie in the shape variability of Keilmesser. In contrast to the distal posterior part, and how it was also noted earlier [8, 13, 14], I could confirm that the working edge is less variable in its convex shape.

In favor of Veil‘s idea of a specific form-function concept, I demonstrated that the mean shape of the highest density within the scatterplot of the two first principal components in shape space (Fig 5) resembles his *ideal* template for the Lichtenberg Keilmesser. In other words, the fixed morphological parts and the relatively similar shapes independent of size may suggest that Neanderthals had a specific concept in mind of how such a tool should have looked like.

However, the analyses of the edge angles draw another picture. Firstly, working edge angles and working edge shape are independent from the varying Keilmesser morphology and Neanderthals tried to keep the specific working edge charcateristics constant during resharpening. In other words, the convex cutting edge was the most important active part of this tool and other tool units had the purpose to keep this functionality alive. I showed that increasing relative thickness of the tools did not commonly result in increased working edge angles. The back and the proximal part of the distal posterior part seem to fulfill foremostly prehensile functions [56–58]. But both were often also modified by majorly coarse retouch into striking platforms to initially shape and later thin the surfaces of the tools (Figs 4 and 12). This design made it possible to create multifunctional edges with angles that enable (1) prehension but also (2) the thinning of the surfaces during initial shaping, and for subsequent working edge angle maintenance [33].

Secondly, the distal posterior part and the back are less standardized in shape and angle than the working edge. For the latter, this might be mainly caused by natural variability as the back often incorporates thick natural and only marginally modified surfaces into the tool design. The distal posterior part was used as a striking platform for thinning (natural or prepared, see Table 2) and Neanderthals took special care in the establishment and maintenance of a pointed or slightly rounded, thin and sharp tip. Therefore, the distal posterior part required three morphological criteria to enable this twofold functionality: (1) large angles at the proximal portion, (2) decreasing angles towards the distal portion, and (3) it needed to be angled towards the working edge to create a tip and to enable thinning of the largest possible volume of the distal tip area. This morphology made removals that vary between directions parallel and perpendicular to the working edge possible. These morphological requirements and the related knapping behavior imply that angle configurations influence the distal tool morphology.

My results do not generally neglect that the manufacture of prehensile parts, striking platforms, a convex cutting edge, and a sharp tip on a tool [12, 13] is related to the idea of a specific form-function concept [8]. But the fracture mechanics and the specific angle configurations that were needed to create this functional units predetermined, in my opinion, its morphological realization. In other words, my data suggests that there may have existed a general template of how a Lichtenberg Keilmesser should look, but “*it seems to be all about the angle*”: For Neanderthals, the goal of these tools was to establish an acute angle along the working edge and the tip, and to maintain these angles during use. In contrast, Neanderthals designed the distal posterior part to have a large edge angle. They realized this with a thick edge, a flat natural surface, intentional breaks, or a fine prepared striking platform. The purpose of the knappers was to establish an edge that they used as a striking platform to thin the distal volume of the tool. This behavior resulted in tools with a rather constant morphology of the working edge and the tip, but a variable distal posterior part. In the course of intensive thinning and (re-) preparation of the edge, the distal posterior part became potentially longer and sharper, causing morphological variability and leading eventually to typologically different but strongly related [8, 13] tool classifications, like Faustkeilblätter.

However, my data also suggests that Keilmesser stayed morphologically Keilmesser: they were ultimately discarded when the main working edge did not fulfill its primary functionality, i.e. when the edge angle increased over 60°. With the data presented here, I could find no evidence that these specific tools were transformed into other tools, e.g., bifacial scrapers [49, 50]. But a future study will analyze the relation between tools further, as I plan to incorporate more tool classes found in the Lichtenberg assemblage, like bifacial and leaf-shaped scrapers.

### The Lichtenberg Keilmesser

Following the results presented here, the Lichtenberg Keilmesser should rather be understood as a dynamic tool concept than a static type. However, the Lichtenberg tool as a type is meaningful in so far that it refers to one solution to create a tool with specific functionalities: cutting, prehension, and reusability. As I explained above, to establish and maintain its functionality, certain angles where created by the knappers along the active edges. This behavior resulted in specific shapes and positions of the active parts and created the *standardized* or *template* morphology of this Keilmesser concept.

Reducing tool shape to edge angle creation and maintenance does not necessarily neglect archaeological groups or *named stone tool industries—NASTIES* [83] that are based on the occurence of specific bifacial tool concepts, like the LMP Keilmessergruppen [7, 8, 12, 13, 48, 66–68, 84] or the contemporaneous Mousterian of Acheulian Tradition (MTA) of western Europe [60]. Recently, Uthmeier [50] argued that the finished tool itself may not have served as social marker for group identity (in the sense of Weißmüller’s [85] finished tools as symbolic markers). He assumes that as tool manufacture and maintenance seems to be learned by social interaction, “[‥] identical or similar manufacture of lithics is another way to confirm that all group members share the same worldview.” (Uthmeier 2016:67). Recently, Frick and Herkert [32] made similar observations for the conceptionally uniform but highly dynamic production of Keilmesser with tranchet blow in their research area Saône-et-Loire, France. In the present case study, the production, resharpening and edge angle maintenance strategy of the Lichtenberg Keilmesser is conceptionally different from these Keilmesser resharpened by a tranchet blow parallel to the distal part of the working edge [30, 32, 48]. So far, I did not observe any clear evidence for the frequent application of the tranchet blow in the assemblages analyzed here, or in neighboring LMP assemblages, like Königsaue or Salzgitter-Lebenstedt, both of which I have analyzed [61, 62]. For the latter, Pastoors [16] reports the occurrence of the technology, but only on three bifacial scrapers and not on Keilmesser. Additionally, he found six flakes resulting from tranchet blows within the assemblage. Furthermore, there are two questionable artifacts from Pouch that may be related to the technique of tranchet blow: the Keilmesser described above and displayed in Fig 4:1 and a flake [19]. However, these examples are single occurences and can therefore not serve as evidences for the frequent application of the tranchet blow as resharpening strategy. In other words, the presence of the Lichtenberg Keilmesser-concept seems mostly to exclude the conceptionally different solution of tranchet blow edge modification and resharpening. This suggests the existence of shared ideas and concepts within a specific Neanderthal life-world or tool manufacture domain. Of course, as the tool only represents a single aspect of Neandertal material culture and daily life [86], the Lichtenberg Keilmesser concept is not necessarily the main marker for identity within a specific Neanderthal realm. But its presence and manufacture strategies might be shared by a late Middle Paleolithic Neanderthal community of yet unknown size, within an estimated geographical range across the northern European Plain from Germany to western Russia [15–18, 20, 22–27] and a time depth between potentially MIS 5a to MIS 3 [28, 84] or MIS 3 only [87].

## Conclusion

Here I have re-analyzed the Lichtenberg Keilmesser. I evaluated the ideas of the Keilmesser as specific form-function concept vs. a pragmatic solution on a tool to maintain edge angles. Using a combined approach of 3DGM and edge angle analysis, I could draw inferences about shape variability, edge morphology variability, edge angle distribution and reduction/resharpening and their influence on shape. The tool consists of two prehensile units, the proximal base and the lateral back, and two active edges, the distal posterior part and the working edge opposite the distal posterior part and the back. The two active edges form together a sharp distal tip. In my analysis, I focus on the two active edges, as the base and the back incorporate natural surfaces to a large extent and are driven by natural variability. However, the distal posterior part was analyzed together with the back, as its morphology and length can only be understood in relation to the latter.

My results show that the morphology of the Lichtenberg Keilmesser is predominantly driven by edge angle configurations to enable the functionality of prehensile and active units on the tool. I could identify two morpho-functional fixed edges, the convex working edge and the sharp distal tip. Especially the working edge has a low variability in shape and angles. This implies that during reduction, resharpening, and reuse, Neanderthals tried to keep these characteristics constant to preserve functionality. An edge angle of 60° is understood as upper threshold for cutting functionality. As the median angles of Keilmesser from the well preserved excavated assemblages Lichtenberg and Pouch only once exceed this threshold by about 1°, I infer that tools might be ultimately discarded if the primary functionality of the working edge could not be maintained. This was further reinforced for my results of the reduction analysis, where I illustrated with two examples successful (the angle was kept low) and unsuccessful resharpening (working edge angle could not be maintained) strategies.

Contrasting the results for the working edge, I found that the second active edge, the distal posterior part, represents a multifunctional edge. Together with the back, it has prehensile functions and serves as a striking platform for surface thinning. Additionally, its distal part was designed as a sharp edge to create a sharp tip together with the working edge. The twofold functionality of the edge was realized by the knappers with specific edge angle configurations: large angles at the proximal portion of the distal posterior part and decreasing angles towards its distal end. The former, i.e. the striking platform, was achieved by coarse or fine preparation, a thick edge, a thick natural surface or an intentional break (Fig 4, Table 2). Subsequent shaping and resharpening of the distal end of Lichtenberg Keilmesser lead to variations in length and shape of the distal posterior part. Therefore, the distal posterior part has a higher range of variability than the working edge and is responsible for a large share of the morphological Keilmesser variability. In conclusion, the distal posterior part can be understood as a unit to maintain the functionality of the two fixed active edges, the working edge and the tip.

Although it seems to be “*all about the angle*”, my analysis does not necessarily argue against a definition of this tool as type. However, this type should not be seen as static, but rather as a dynamic concept. I understand the Lichtenberg Keilmesser as conceptual solution to create and maintain certain functional purposes with the help of specific edge angle configurations. The morphological requirements for these configurations result in shape characteristics that we today identify and recognize as a type or a form-function concept. However, I cannot rule out that Neanderthals had a template in mind for the positioning of prehensile and active parts when they manufactured a unifacially shaped or bifacial Lichtenberg Keilmesser.

## Supporting information

S1 File (ZIP)

S1 Table (CSV)
