# Supplementary figures and images for: The Lichtenberg Keilmesser - it’s all about the angle
Source: PLoS One. 2020 Oct 6;15(10):e0239718. doi: 10.1371/journal.pone.0239718 (PMC7538202; doi:10.1371/journal.pone.0239718)

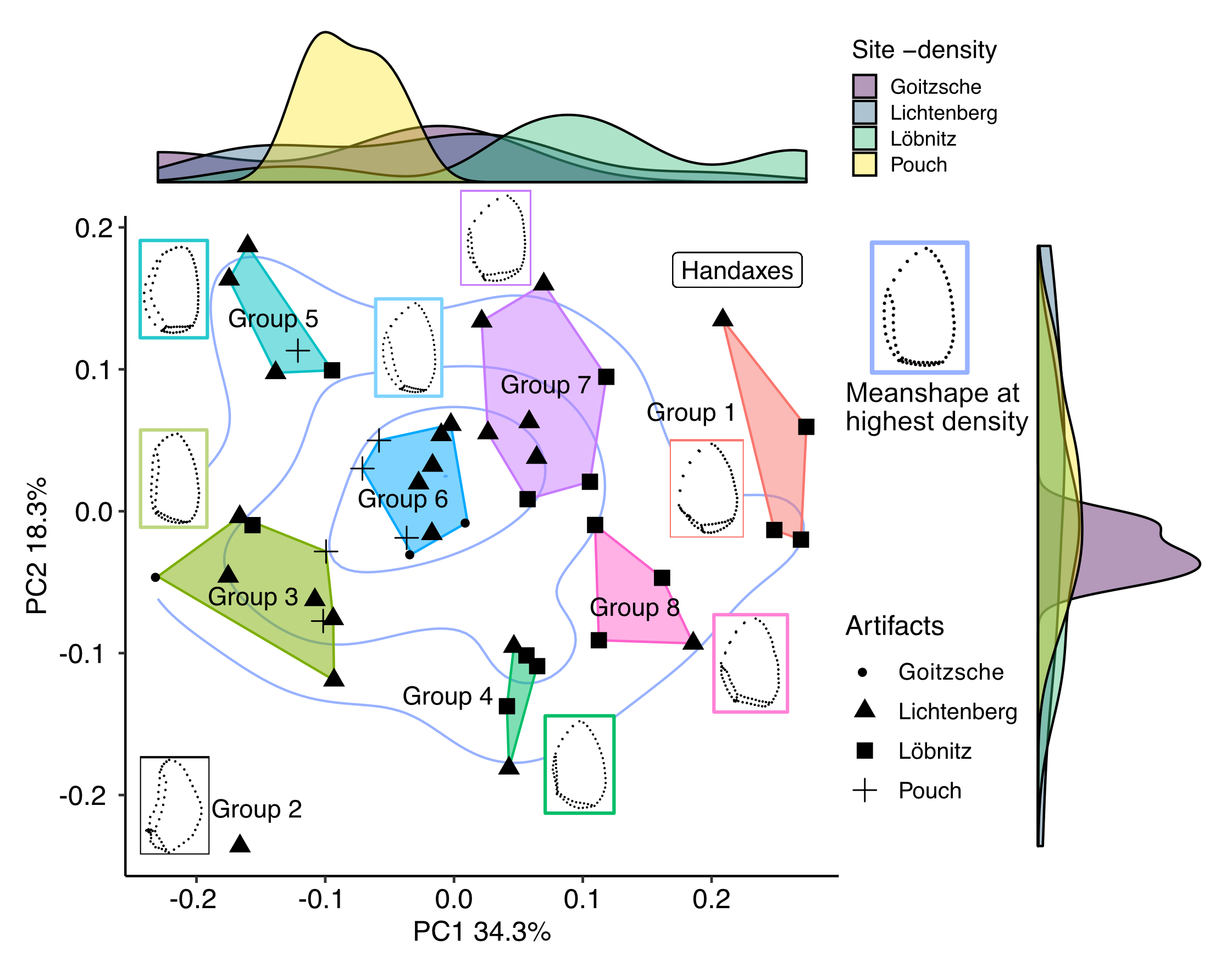

Supplement: S1 File — (ZIP) [file pone.0239718.s001.zip › SI_Compile_Article_M_Weiss/Fig5.jpg]

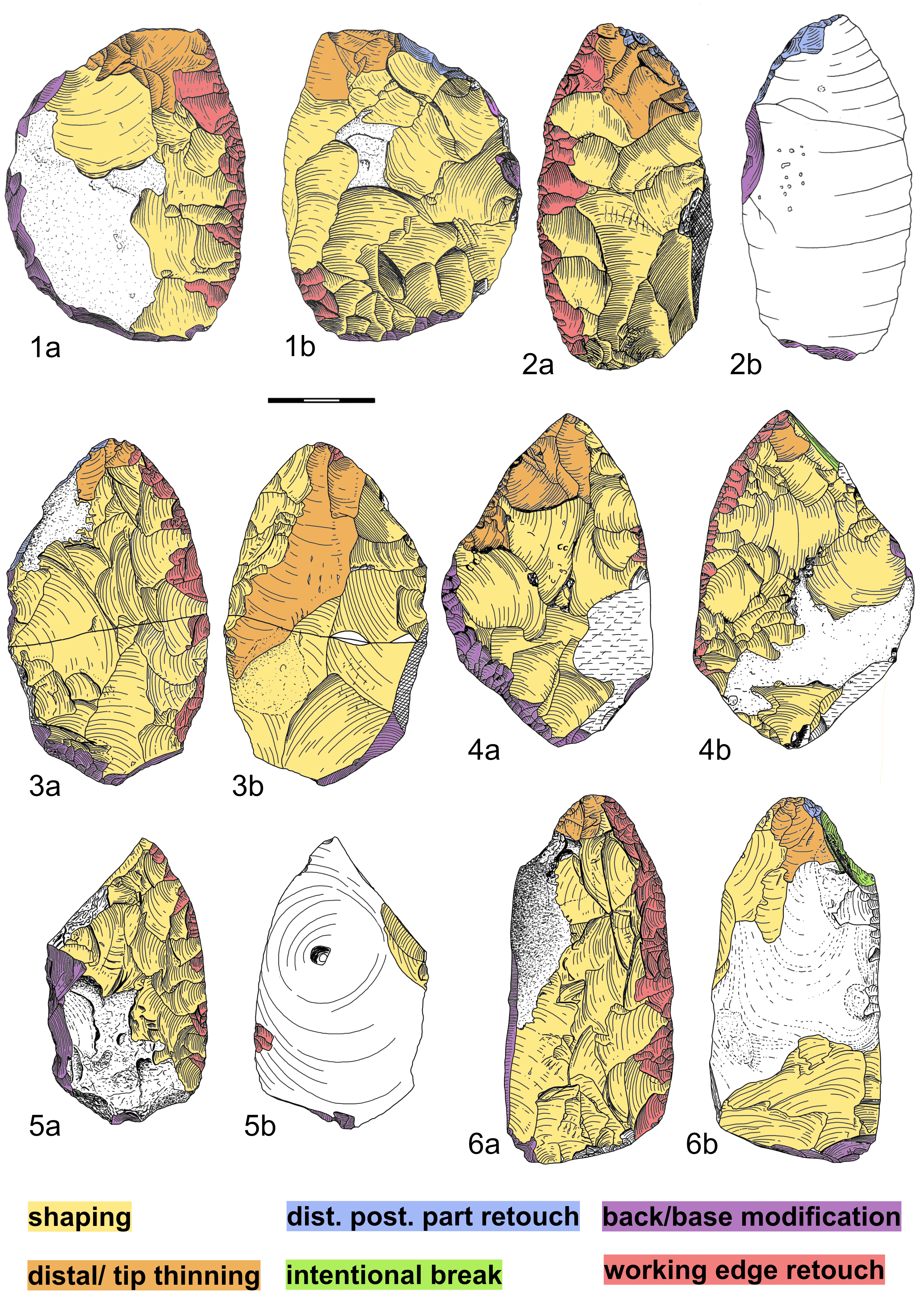

Supplement: S1 File — (ZIP) [file pone.0239718.s001.zip › SI_Compile_Article_M_Weiss/Fig4.jpg]

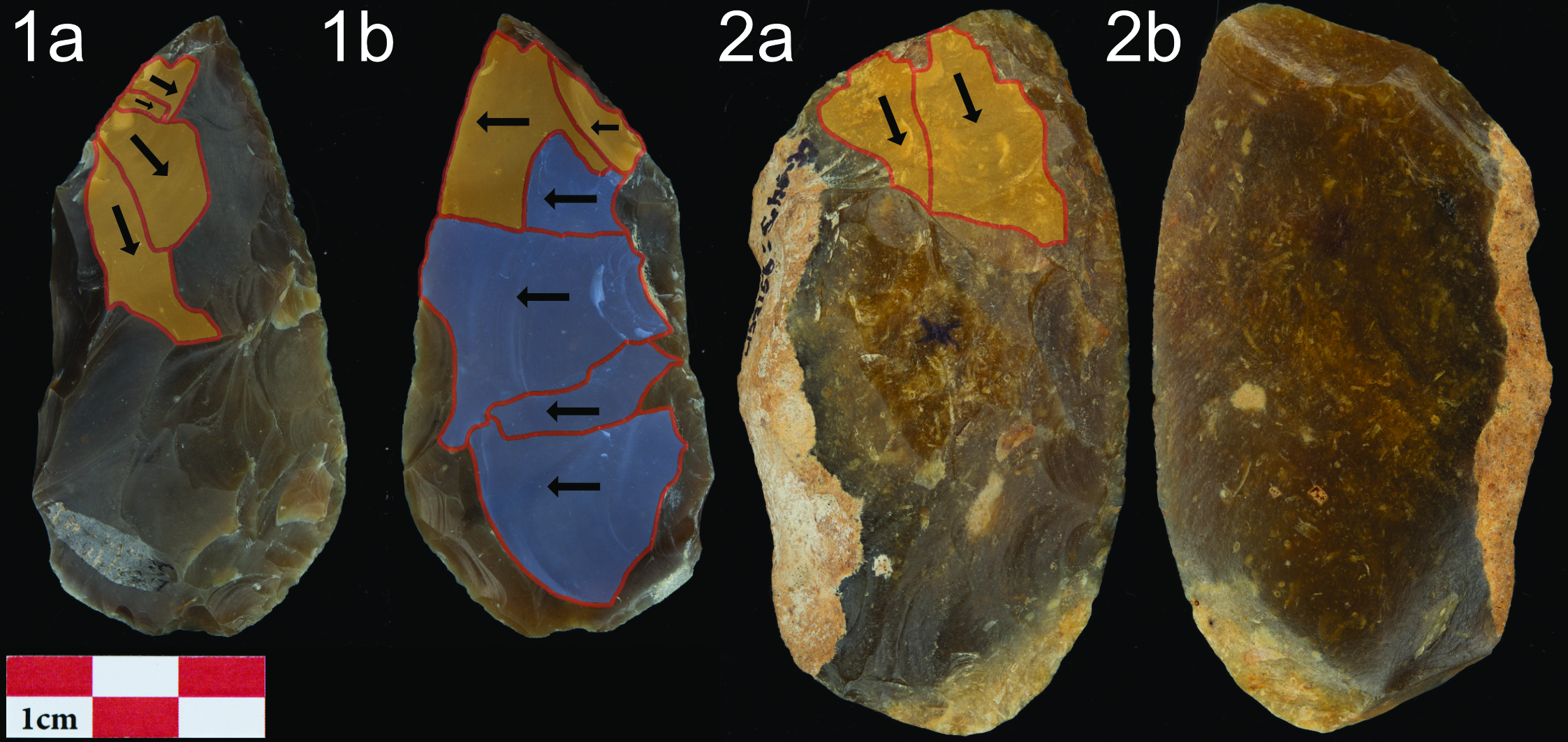

Supplement: S1 File — (ZIP) [file pone.0239718.s001.zip › SI_Compile_Article_M_Weiss/Fig12.jpg]

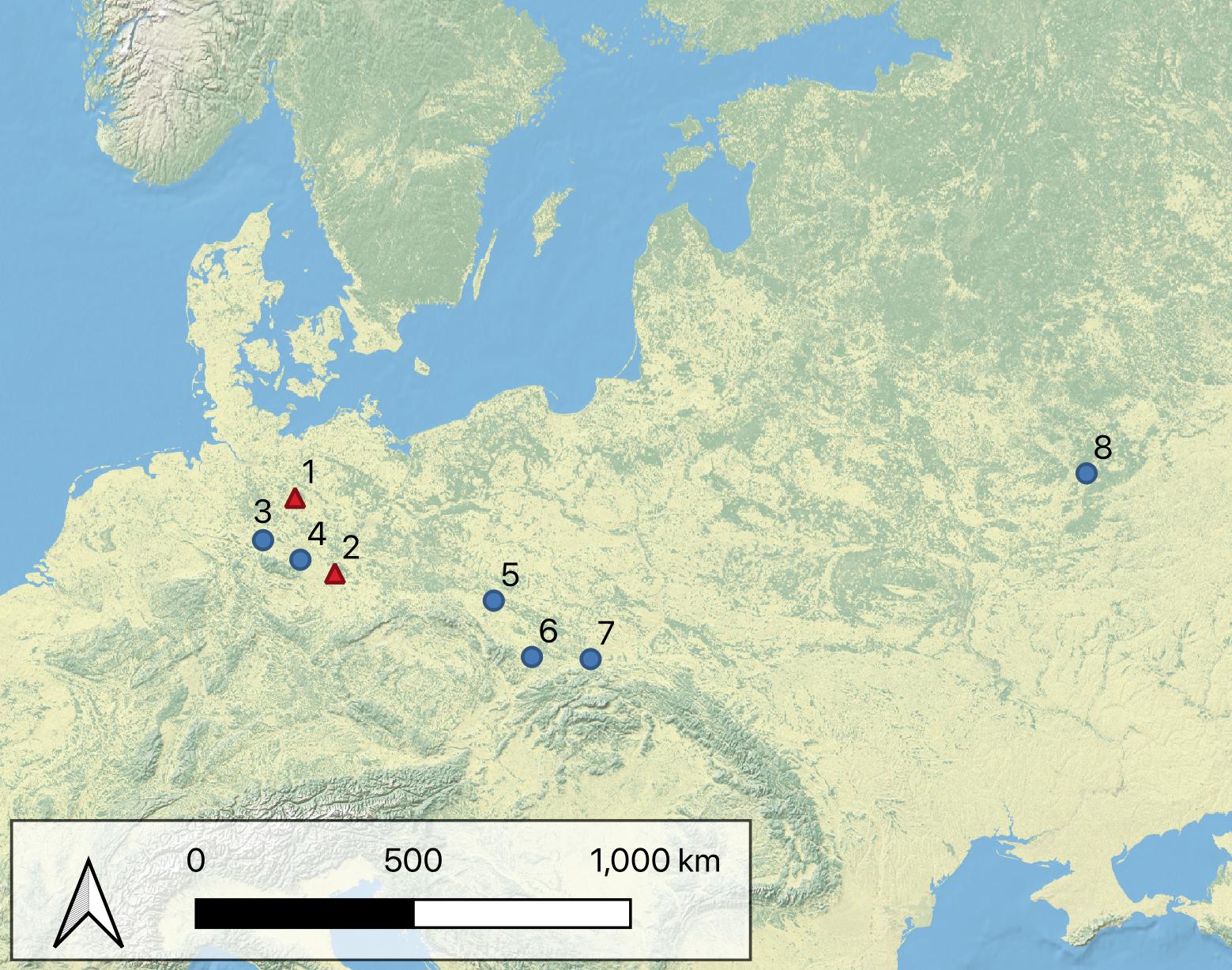

Supplement: S1 File — (ZIP) [file pone.0239718.s001.zip › SI_Compile_Article_M_Weiss/Fig1.jpg]

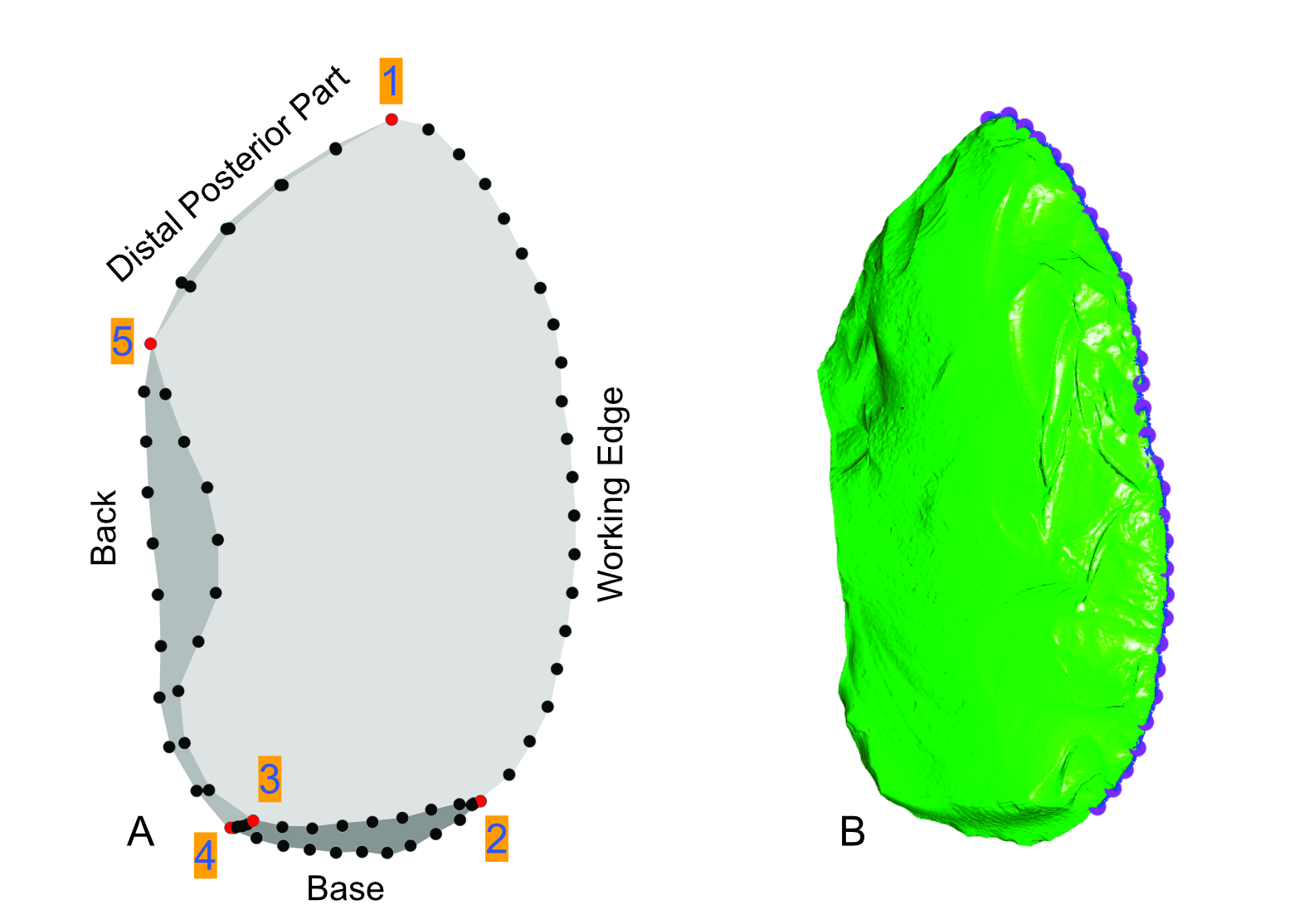

Supplement: S1 File — (ZIP) [file pone.0239718.s001.zip › SI_Compile_Article_M_Weiss/Fig3.jpg]

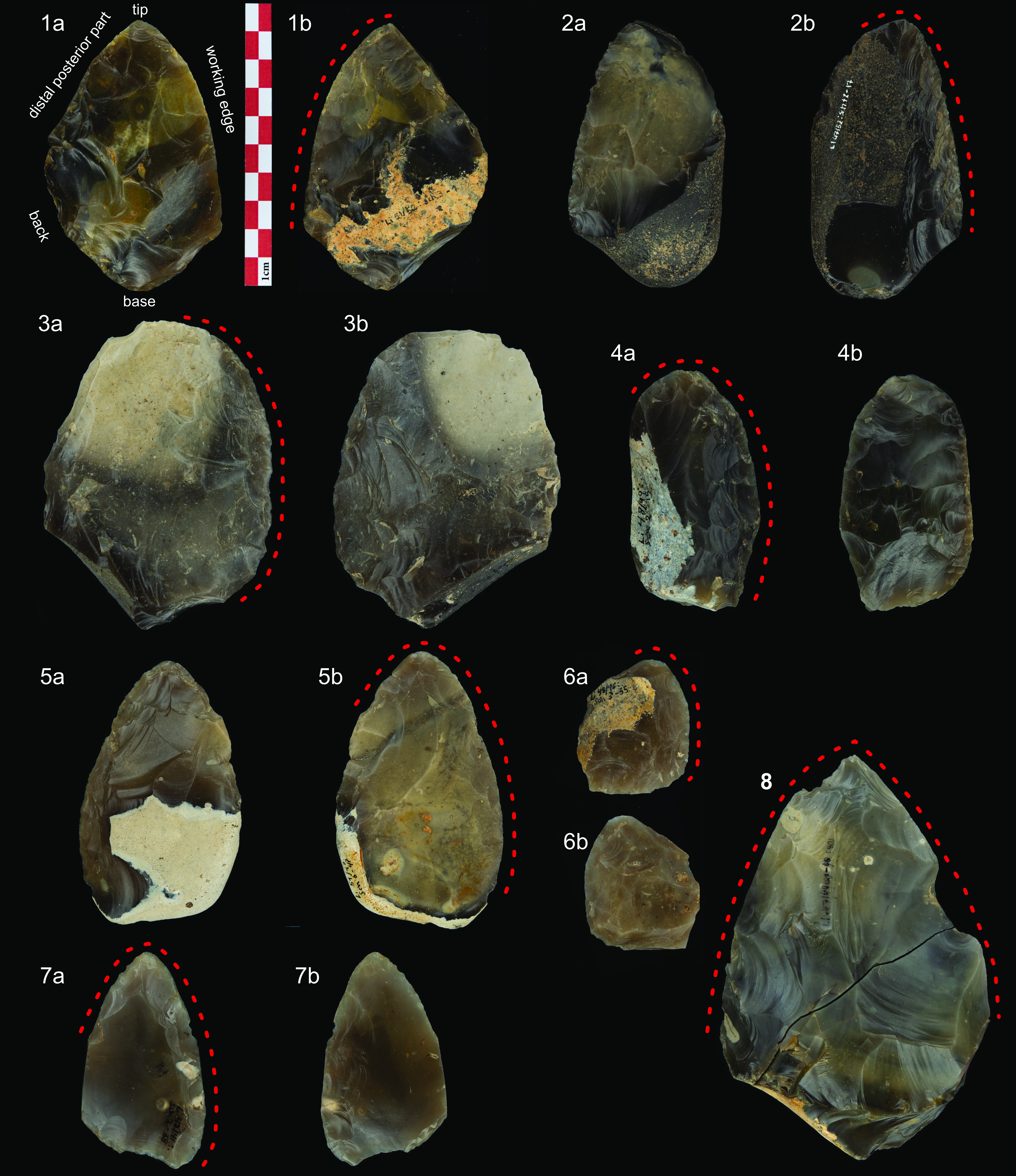

Supplement: S1 File — (ZIP) [file pone.0239718.s001.zip › SI_Compile_Article_M_Weiss/Fig2.jpg]

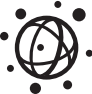

**PLOS**

**SUBMISSION**

Supplement: S1 File — (ZIP) [file pone.0239718.s001.zip › SI_Compile_Article_M_Weiss/PLOS-submission-eps-converted-to.pdf]
